# Therapeutic potential and underlying mechanisms of engineered young plasma-derived exosomes in Alzheimer's disease

**DOI:** 10.1016/j.bioactmat.2026.08.008

**Published:** 2026-08-15

**Authors:** Hang Chen, Yuanquan Si, Qian Cheng, Qian Yu, Zhikang Cui, Shuyi Yu, Xiaoyi Zhao, Yan Jin, Yunshan Wang, Ming Li, Zhiming Lu

**Affiliations:** Department of Clinical Laboratory, Shandong Provincial Hospital Affiliated to Shandong First Medical University, Jinan, Shandong, 250021, China

**Keywords:** Alzheimer's disease, Exosome, *RPTOR*, Autophagy

## Abstract

Exosomes (EXOs) derived from the plasma of young individuals are believed to have the potential to ameliorate aging-related memory deficits. However, their specific roles and mechanisms in Alzheimer's disease (AD) therapy have not yet been systematically investigated. In this study, the rabies virus glycoprotein-targeting peptide (RVG-29) was conjugated to the surface of young plasma-derived EXOs to construct RVG-engineered EXOs (RVG-EXOs), and their therapeutic potential and underlying mechanisms in AD models were systematically evaluated. In 3×Tg AD model mice, exogenous administration of young plasma-derived EXOs and their engineered product (RVG-EXOs) revealed that RVG-EXOs could more efficiently enter brain tissue and target neurons, significantly reduce Aβ plaque and phosphorylated Tau (P-Tau) pathological deposition, restore synaptic structure, promote neuronal survival, and improve cognitive behavior. Mechanistic studies demonstrated that RVG-EXOs inhibited *RPTOR* expression, thereby activating the autophagy pathway and promoting the clearance of pathological proteins. Both in vitro and in vivo experiments confirmed that overexpression of *RPTOR* significantly suppressed the therapeutic effects of RVG-EXOs. single-cell transcriptomic profiling further revealed that RVG-EXOs not only increased neuronal proportion and modulated excitatory/inhibitory neuronal balance but also reshaped the microglial landscape by reducing deleterious disease-associated while increasing homeostatic surveillant microglia. In summary, this study not only reveals for the first time the potential value of young plasma-derived EXOs in AD treatment but also, through RVG engineering strategies and the elucidation of the *RPTOR*-autophagy mechanism, provides new insights for targeted therapy of neurodegenerative diseases.

## Highlights

• RVG-engineered young plasma-derived exosomes (RVG-EXOs) were first constructed, enhancing blood–brain barrier penetration and neuron targeting, thus offering an efficient delivery system for targeted AD therapy.
• RVG-EXOs treatment markedly improved multiple cognitive behaviors in 3×Tg AD mice, including spatial learning, working memory, and novel object recognition.
• RVG-EXOs effectively alleviated AD pathology by promoting Aβ clearance, suppressing Tau hyperphosphorylation, and preserving synaptic integrity and neuronal survival.
• Mechanistic studies revealed that RVG-EXOs activates the autophagy pathway by inhibiting *RPTOR* expression, providing a molecular basis for the neuroprotective effects of young plasma exosomes.
• Single-cell RNA sequencing revealed that RVG-EXOs remodeled brain cellular landscape: increased neurons, rebalanced inhibitory/excitatory neurons, strengthened Ptn–Sdc3 neuroprotection, and attenuated APP–CD74 signaling, which suppressed disease-associated microglia (DAM) and promoted homeostatic microglia.

## Graphical abstract

**Figure:**
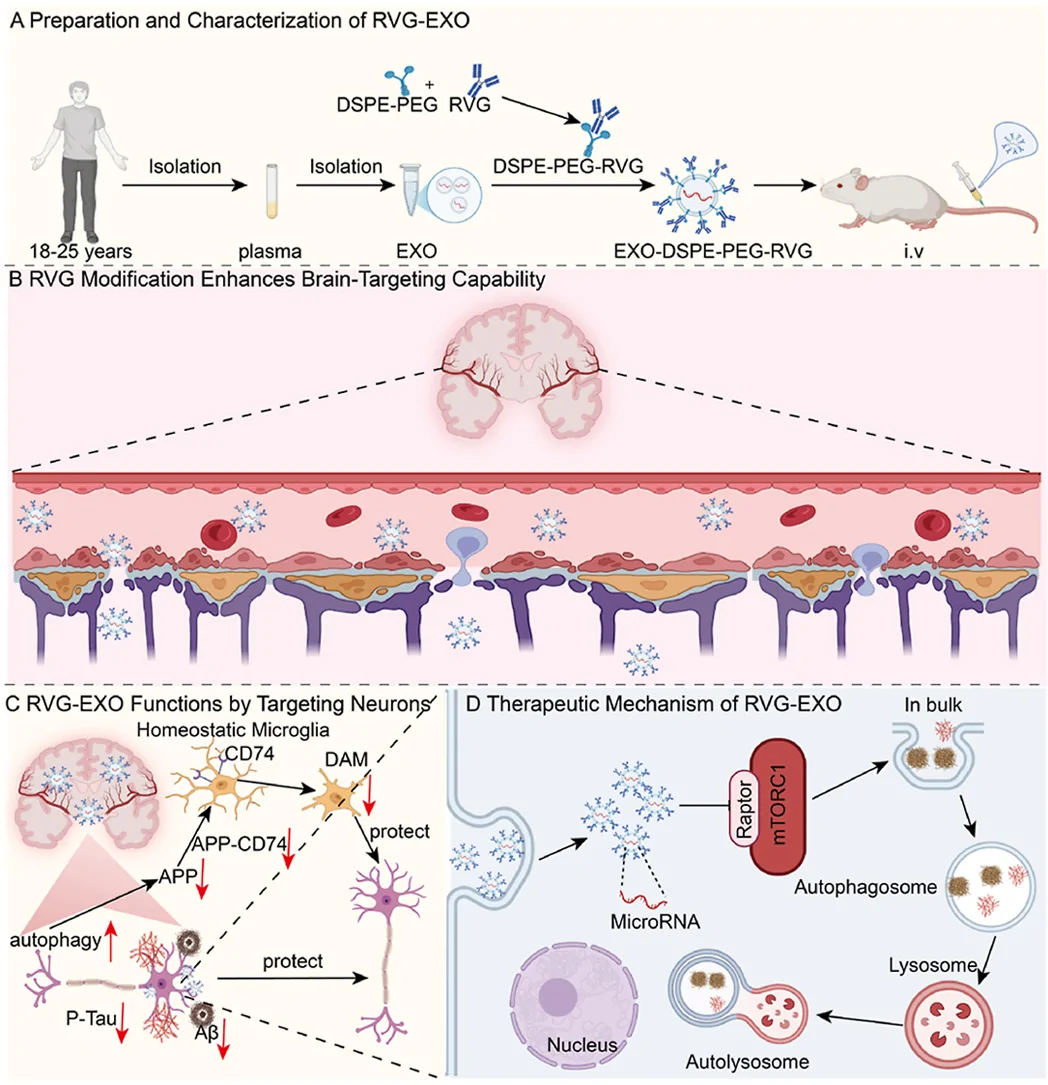

## 1 Introduction

Alzheimer's disease (AD) is a neurodegenerative disorder characterized primarily by progressive cognitive dysfunction and memory decline, and its onset and progression are closely associated with the aging process. Its core pathological features include abnormal deposition of amyloid-β (Aβ) plaques and the formation of neurofibrillary tangles composed of hyperphosphorylated microtubule-associated protein, phosphorylated Tau (P-Tau) [1]. The accumulation of these pathological proteins leads to synaptic damage, neuronal loss, and neuroinflammation, ultimately resulting in severe cognitive deficits [2]. Currently, clinical treatment of AD primarily relies on cholinesterase inhibitors and N-methyl-D-aspartate (NMDA) receptor antagonists to alleviate symptoms in patients with moderate to severe disease, but it cannot stop or reverse the disease progression [3]. In recent years, monoclonal antibodies targeting Aβ (such as aducanumab and lecanemab) have been approved for early-stage AD treatment, demonstrating disease-modifying potential. However, they still face challenges such as high cost, risk of side effects, and limited efficacy [4,5]. Overall, existing therapies are fundamentally limited by their inability to effectively cross the blood-brain barrier (BBB), difficulty in clearing pathological proteins already formed, incapacity to repair damaged neurons, and relatively late treatment windows. Therefore, developing novel therapeutic strategies that are precisely targeted, effectively intervene, and possess high safety remains a critical challenge awaiting breakthrough.

Exosomes (EXOs) are nano-sized vesicles actively secreted by cells, carrying bioactive substances such as proteins and nucleic acids. Due to their inherent biocompatibility, low immunogenicity, and potential to cross biological barriers, they have emerged as promising natural carriers in the fields of drug delivery and disease therapy [6]. In recent years, EXO-based therapeutic strategies have shown unique promise for age-related diseases, particularly neurodegenerative disorders such as AD. Ashok et al. utilized EXOs engineered with Fe65 and loaded with the compound corynoxine-B to significantly enhance autophagy function and improve cognitive behavior in AD model animals [7]. Another study found that hypoxia-preconditioned mesenchymal stem cell-derived EXOs could ameliorate synaptic dysfunction, modulate the inflammatory response in APP/PS1 mice, and mitigate cognitive decline [8].

To date, the role of blood-derived EXOs from young individuals in reversing age-related functional decline has attracted significant attention. Studies have shown that plasma-derived EXOs from young individuals demonstrate potential in ameliorating age-related functional decline and memory deficits [9]. Similarly, young serum-derived EXOs have also been shown to ameliorate age-related cognitive decline in aged mice [10]. In contrast to young EXOs, EXOs secreted by senescent cells may carry and transmit senescence-associated secretory phenotype components, such as pro-inflammatory factors, reactive oxygen species, and dysregulated microRNAs (miRNAs), thereby exacerbating neuroinflammation, oxidative stress, and cellular dysfunction, potentially promoting the progression of AD pathology [11]. Although young plasma-derived EXOs have attracted significant attention in the field of anti-aging, their direct therapeutic efficacy in AD treatment, the synergistic amplification effect following engineering modification, and the underlying molecular mechanisms have not yet been systematically elucidated.

Furthermore, natural EXOs inherently suffer from poor in vivo targeting ability and difficulty in accumulating at specific lesion sites (such as the brain), greatly limiting their therapeutic application [12]. The rabies virus glycoprotein (RVG)-derived peptide (RVG-29) can specifically recognize acetylcholine receptors on the neuronal surface and has been demonstrated to effectively mediate carriers in crossing the BBB and targeting neurons [13]. To enhance the delivery efficiency of young plasma-derived EXOs to neurons, this study performed surface engineering modifications on these EXOs. The targeting peptide RVG-29, which can specifically recognize neuronal surface receptors, was selected and conjugated onto the surface of young plasma EXOs via a lipid-anchoring method to construct RVG-EXOs.

Autophagy is a critical intracellular pathway involved in the clearance of misfolded proteins and damaged organelles, and its dysfunction has been closely linked to the pathogenesis of various neurodegenerative diseases, including AD [7,14,15]. Mammalian target of rapamycin complex 1 (mTORC1) serves as a key negative regulator of autophagy, and regulatory-associated protein of mTOR (RPTOR), also known as Raptor, as the core scaffold protein of mTORC1, plays a central role in autophagy regulation by mediating the inhibitory effect of mTORC1 on the ULK1 autophagy initiation complex [16]. Recent studies have suggested a potential association between RPTOR and the pathological progression of AD [17]. However, whether RPTOR can serve as a molecular target for exosome-based therapy has not yet been reported. The goals of our study were to: (1) using an AD cell model and a 3xTg transgenic mouse model to evaluate the brain delivery efficiency and neuronal targeting capability of RVG-EXOs, and analyze their effects on Aβ deposition, P-Tau levels, and the cognitive behavior of mice; (2) employing molecular biology approaches to investigated the mechanisms by which RVG-EXO intervention promotes the clearance of pathological proteins and exerts neuroprotective effects.

## 2 Results

### 2.1 Preparation, characterization, and functional validation of RVG-EXOs for entering brain tissue and targeting neurons

**Fig. 1:**
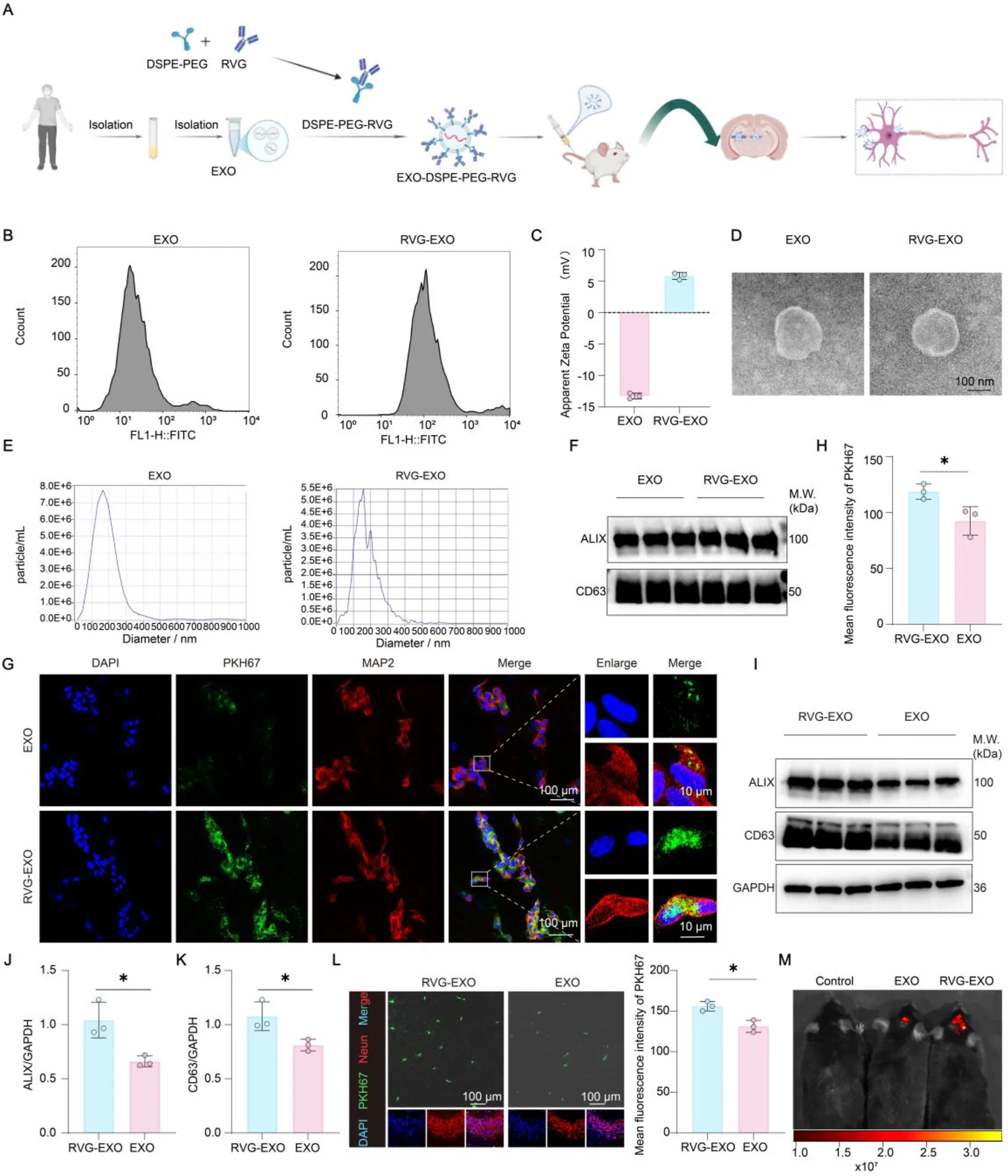
Preparation, characterization, and functional validation of RVG-EXOs for entering brain tissues and targeting neurons. **(A)** Schematic diagram of the RVG-EXO production. **(B)** Flow cytometry histograms showing FITC fluorescence intensities of EXOs and RVG-EXOs. **(C)** Zeta potential of EXOs and RVG-EXOs. Data are presented as mean ± SD (n = 3). **(D)** Representative TEM images of EXOs and RVG-EXOs. **(E)** Size distribution profiles of EXOs and RVG-EXOs determined by nanoparticle tracking analysis. **(F)** Western blot analysis of the exosomal marker proteins ALIX and CD63 in EXOs and RVG-EXOs. **(G)** Representative immunofluorescence images and corresponding quantitative analysis **(H)**, showing the targeting of PKH67-labeled EXOs and RVG-EXOs to SH-SY5Y cells. Data are presented as mean ± SD (n = 3). Statistical significance is determined by the Student's t-test based on *P* < 0.05 (*). **(I–K)** Western blot analysis and corresponding quantitative analysis of exosomal marker proteins (ALIX and CD63) in SH-SY5Y cells after 24-h co-culture with RVG-EXOs or EXOs. Data are presented as mean ± SD (n = 3). Statistical significance is determined by the Student's t-test based on *P* < 0.05 (*). **(L)** Representative immunofluorescence images and quantitative analysis of the hippocampal CA3 region in mouse brain tissues 4 h after intravenous tail vein injection of PKH67-labeled RVG-EXOs or EXOs. Data are presented as mean ± SD (n = 3). Statistical significance is determined by the Students' t-test based on *P* < 0.05 (*). **(M)** In vivo imaging in mice with DiR-labeled RVG-EXOs or EXOs.

To construct engineered EXOs with enhanced brain-targeting capability, plasma was collected from young individuals aged 18–25 years and isolated plasma-derived EXOs. First, 1,2-distearoyl-sn-glycero-3-phosphoethanolamine-polyethylene glycol 2000 (DSPE-PEG2000) was reacted with RVG-29 to generate DSPE-PEG2000-RVG29 nanomicellar particles, which were then co-incubated with the isolated young plasma-derived EXOs to obtain EXO-DSPE-PEG2000-RVG29 (Fig. 1A). Then, nanoscale flow cytometry detection was performed on ordinary young plasma-derived EXOs and RVG-EXOs using FITC-conjugated RVG antibody. The results showed that the mean fluorescence intensity (MFI) of RVG-EXOs was 387, whereas the MFI of ordinary EXOs was only 61.7, indicating that the FITC fluorescence signal intensity of RVG-EXOs was approximately 6-fold higher than that of ordinary EXOs (Fig. 1B and S1A). Since RVG29 carried a positive charge, if it was successfully conjugated to the EXO surface, the membrane potential of the EXOs would shift. Therefore, the membrane potentials of ordinary young plasma-derived EXOs and RVG-EXOs were measured. The results showed that the membrane potential of RVG-EXOs reached approximately 5.21 (Fig. 1C). Together, these results demonstrated that the RVG peptide was successfully anchored onto the EXOs surface and maintained a high modification intensity. Transmission electron microscopy (TEM) images of ordinary EXOs and RVG-EXOs demonstrated that the morphology of EXOs remained intact after engineering modification of the EXO surface with RVG (Fig. 1D). Nano-tracking analyzer (NTA) results showed that the average size distribution of ordinary young plasma-derived EXOs and RVG-EXOs was predominantly around 180 nm (Fig. 1E). Quantitative analysis revealed that the concentration of isolated EXOs was approximately 7.8 × 10^6^ particles/μL, while RVG-EXOs yielded approximately 5.5 × 10^6^ particles/μL after engineering and purification (Fig. 1E). This yield was within the typical ranges reported for plasma-derived EXOs using commercial precipitation-based kits [18,19]. Western blot analysis showed that after RVG engineering, exosomal marker proteins such as CD63 and ALIX were still detected (Fig. 1F). To further assess the purity of the isolated EXOs, we examined the expression of the negative markers GM130 (a Golgi marker) and Calnexin (an endoplasmic reticulum marker). Western blot analysis revealed that neither GM130 nor Calnexin bands were detected in the EXOs and RVG-EXOs samples, whereas distinct bands were observed in the cell lysate (positive control) (Fig. S1B). These results indicated that the isolated EXOs samples were largely free of contamination from Golgi and endoplasmic reticulum sources, suggesting relatively high purity.

To evaluate the neuronal targeting capability of RVG-EXOs, equal amounts of PKH67-labeled EXOs and RVG-EXOs were co-cultured with SY5Y cells of the same concentration for 24 h, followed by immunofluorescence staining. The results indicated that, compared with the EXO group, the number of RVG-EXOs entering the cells was significantly increased (Fig. 1G and H). Subsequently, the equal amounts of EXOs and RVG-EXOs were we co-cultured with SY5Y cells of the same concentration for 24 h and then subjected to Western blot analysis. The results showed that, compared with the EXO group, the level of exosomal marker proteins in the RVG group was significantly increased (Fig. 1I–K). These results collectively demonstrated that RVG-EXOs possessed an enhanced ability to target neurons. Effective treatment of AD required successful entry of the medicines into the brain. To examine the ability of RVG-EXOs to enter brain tissue, PKH67-labeled control EXOs and RVG-EXOs were administered to 3xTg mice via tail vein injection. In 4 h, brain tissues were collected for fluorescence staining. Notably, the fluorescence intensity of PKH67-labeled RVG-EXOs in the brain tissue of 3xTg mice was significantly higher than that of control EXOs (Fig. 1L). Additionally, longitudinal in vivo imaging of DiR-labeled EXOs or RVG-EXOs revealed that, compared with ordinary young plasma-derived EXOs, RVG-EXOs exhibited significantly higher level of accumulation in the brain (Fig. 1M). These results indicated that RVG engineering effectively enhanced the delivery of EXOs to brain tissue. In summary, the above results demonstrated that RVG engineering significantly enhanced the ability of young plasma-derived EXOs to target neurons and to be delivered to brain tissue.

Next, this study employed small animal in vivo imaging to investigate the biodistribution of EXOs and RVG-EXOs in major organs throughout the mouse body, as well as their duration and proportion in brain tissue. The results showed that EXOs and RVG-EXOs were mainly enriched in organs such as the brain, heart, liver, and kidney. Compared with EXOs, the distribution of RVG-EXOs in brain tissue was significantly increased (Fig. S1C). To reflect the proportion of EXOs and RVG-EXOs in brain tissue, we calculated the fluorescence intensity in brain tissue as a percentage of the total fluorescence intensity in all major target organs. The results showed that the proportions of EXOs and RVG-EXOs in brain tissue were approximately 21% and 40%, respectively (Fig. S1C). Small animal in vivo imaging showed that at 6 h, the presence of EXOs and RVG-EXOs was detected in brain tissue; at 24 h, the highest level of distribution of EXOs and RVG-EXOs in brain tissue was detected; and at 48 h, EXOs and RVG-EXOs were almost not detected in brain tissue (Fig. S1C). This indicated that EXOs and RVG-EXOs could maintain in brain tissue for approximately 48 h. Since RVG-EXOs exerted their corresponding effects mainly by targeting neurons after entering brain tissue, this study further investigated the uptake of EXOs and RVG-EXOs by neurons in vivo. PKH67-labeled control EXOs and RVG-EXOs were administered to mice via tail vein injection. In 24 h, brain tissues were collected for fluorescence staining. Immunofluorescence staining results showed that both EXOs and RVG-EXOs could be taken up by neurons in brain tissue, and RVG engineering modification significantly enhanced the uptake capacity of EXOs by neurons (Fig. S1D).

### 2.2 RVG-EXOs improve cognitive behaviors in 3xTg mice

**Fig. 2:**
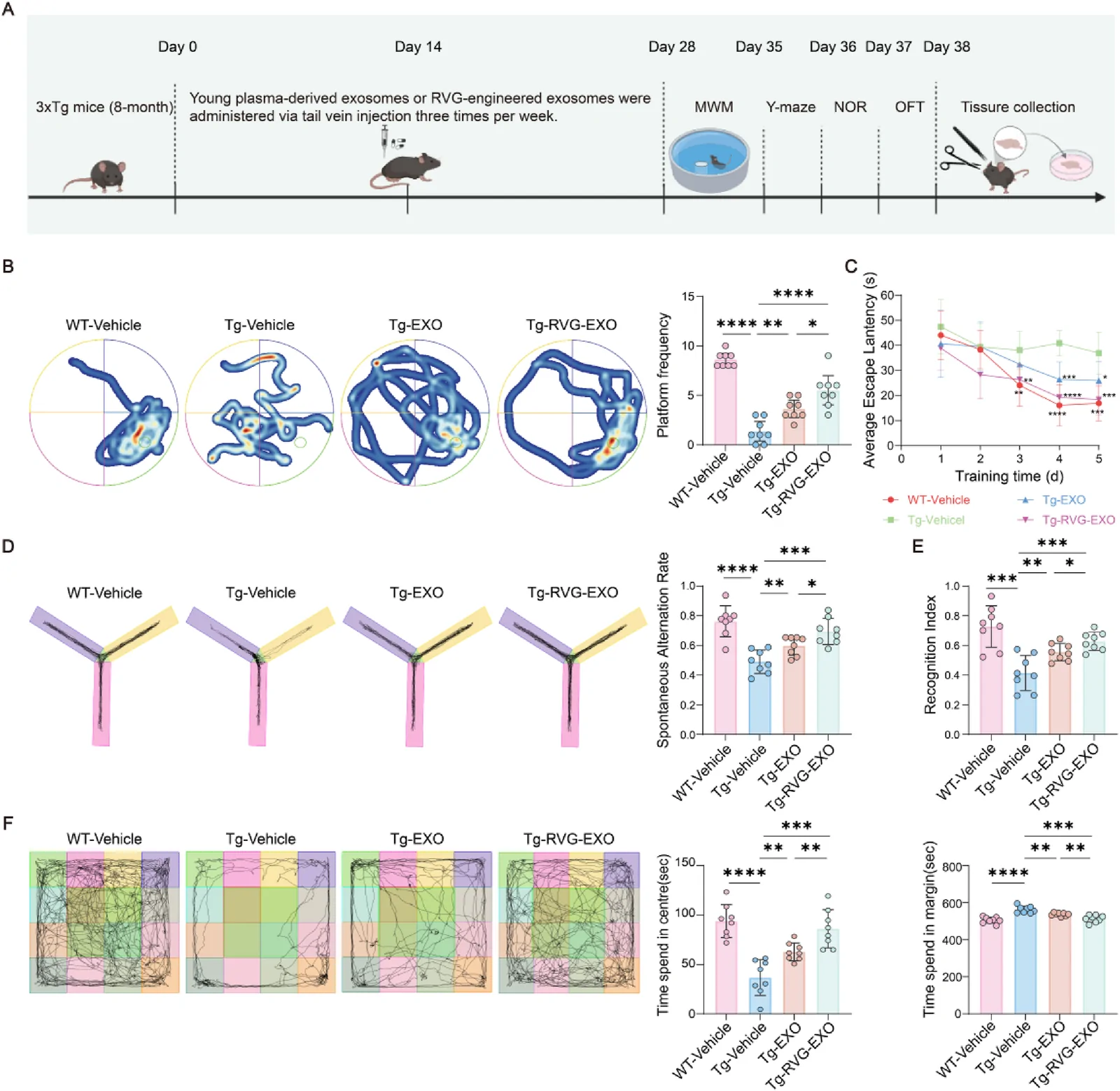
RVG-EXOs improve cognitive behaviors in 3xTg mice. **(A)** Experimental flowchart. **(B)** Representative trajectory heatmaps based on the MWM probe test and quantification of the number of platform crossings (n = 8). **(C)** Representative plots of escape latency in the MWM test (n = 8). Data are presented as mean ± SD. Statistical significance is determined by the Student's t-test based on *P* < 0.05 (*), *P* < 0.01 (**), *P* < 0.001 (***), and *P* < 0.0001 (****), compared to the WT-Vehicle group. **(D)** Representative activity trajectories in the Y-maze and quantitative analysis of the spontaneous alternation rate (n = 8). **(E)** Recognition index in the NOR test (n = 8). **(F)** Representative OFT tracks and analysis of center versus periphery time (n = 8). Data are presented as mean ± SD (n = 8). Statistical significance is determined by the Student's t-test based on *P* < 0.05 (*), *P* < 0.01 (**), *P* < 0.001 (***), and *P* < 0.0001 (****).

Studies have indicated that the bioactivity of EXOs exhibits almost no species specificity, i.e., young human plasma-derived EXOs can exert effects comparable to those of young mouse plasma-derived EXOs [9,20,21]. To investigate the effects of RVG-EXOs on cognitive impairment in 3xTg mice, the mice were divided into different groups for intervention, followed by a series of behavioral tests, including the Morris Water Maze (MWM), Y-maze, Novel Object Recognition (NOR), and Open Field Test (OFT) (Fig. 2A).

First, the effects of RVG-EXOs on the spatial learning and memory abilities of the mice were evaluated using the MWM. On the first day, the mice underwent visual training to assess their vision and swimming ability. Subsequently, hidden platform training was conducted over 5 d for the different groups of mice. On the 7th day, the platform was removed, and a probe test was performed using animal tracking software to assess the memory function of the mice. Notably, during the hidden platform training, compared to the negative control mice, the 3xTg mice injected with saline exhibited slower learning ability and longer escape latencies during the learning process, indicating impaired learning ability in the 3xTg mice. In contrast, compared to the positive control mice, the 3xTg mice treated with either RVG-EXOs or EXOs demonstrated faster learning capability and shorter escape latencies, with more pronounced effect detected in the RVG-EXO-treated group. These results suggested that both RVG-EXOs and EXOs could improve the learning ability of 3xTg mice, with RVG-EXOs having a more significant effect. In the subsequent probe test, compared to the negative control mice, the untreated 3xTg mice crossed the target platform significantly fewer times. However, after treatment with RVG-EXOs or EXOs, the number of times that the 3xTg mice crossed the target platform was increased significantly, with more evident effect revealed in the RVG-EXOs-treated group. Overall, the MWM results indicated that both RVG-EXOs and EXOs could improve the learning and memory abilities of 3xTg mice, with RVG-EXOs exhibiting a stronger effect (Fig. 2B and C).

Next, the short-term spatial working memory of the mice was assessed using the Y-maze. Compared with the negative control group, the spontaneous alternation rate of mice in the positive control group was significantly decreased, indicating an impairment in short-term spatial working memory. Treatment with either RVG-EXOs or EXOs could partially improve this impairment, with more pronounced effect detected in the RVG-EXO-treated group (Fig. 2D).

Subsequently, the NOR test was performed on the mice. The results showed that, compared with the negative control mice, the recognition index (RI) of the positive control mice was significantly reduced, indicating impaired short-term object recognition memory in the 3xTg mice. Treatment with either RVG-EXOs or EXOs could partially restore the object recognition memory of the mice, with RVG-EXOs demonstrating a stronger effect (Fig. 2E). Finally, the OFT was performed to assess anxiety-like behavior in the mice. Notably, after treatment with either RVG-EXOs or EXOs, the time spent in the central area by 3xTg mice was significantly increased, with the more pronounced effect revealed in the RVG-EXO-treated group (Fig. 2F). These results indicated that both RVG-EXO and EXO treatments could alleviate anxiety-like behavior and improve cognitive function in 3xTg mice, with RVG-EXO exhibiting a stronger effect. Overall, RVG-EXO treatment improved various cognitive behaviors in 3xTg mice.

### 2.3 RVG-EXOs promote the clearance of pathological proteins such as Aβ and P-Tau and protect neurons

**Fig. 3:**
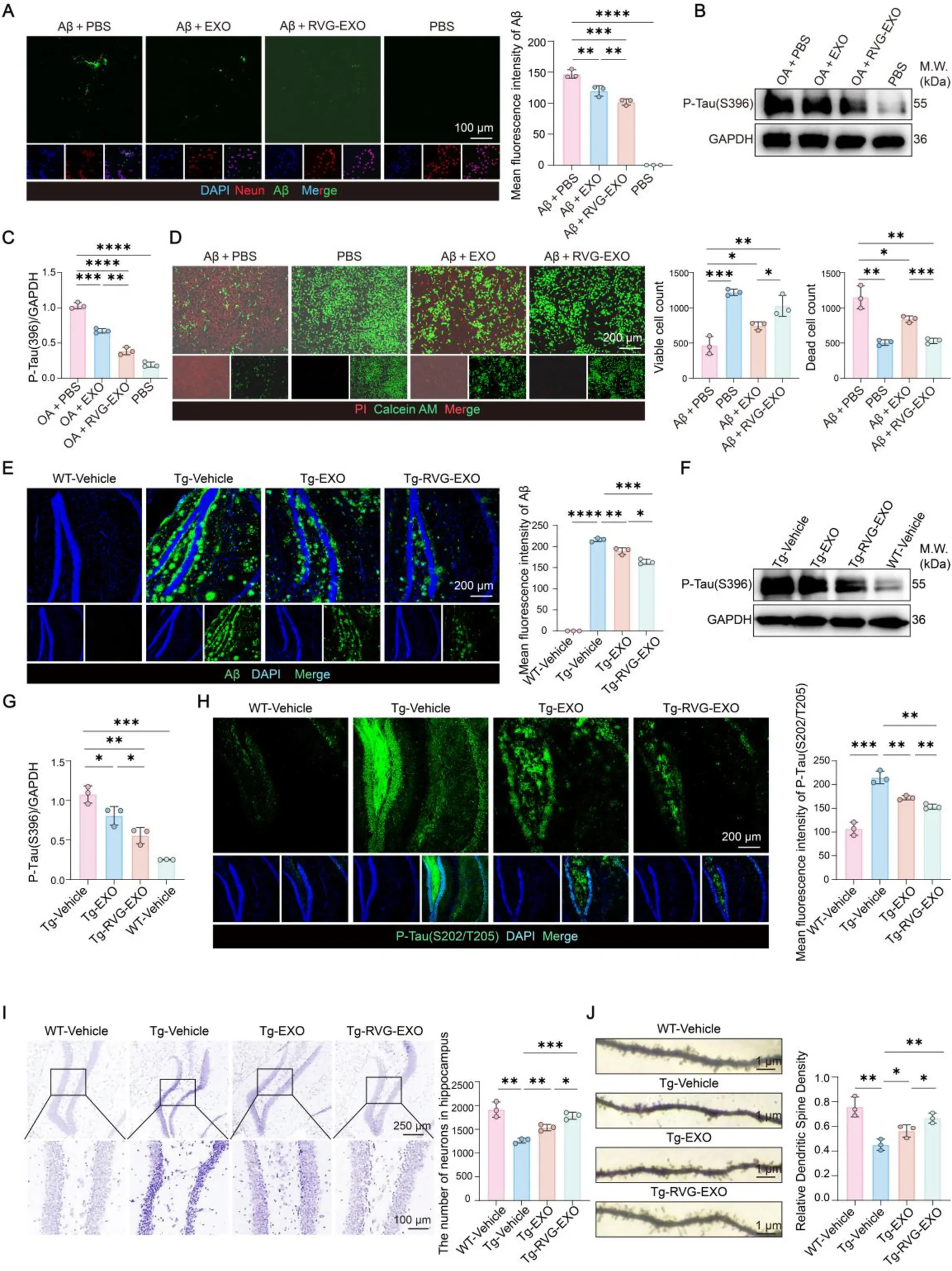
RVG-EXOs promote the clearance of pathological proteins such as Aβ and P-Tau both in vitro and in vivo and protect neurons. **(A)** Representative immunofluorescence images and quantitative analysis of 647-Aβ1-42 (n = 3). Green, 647-Aβ1-42; red, Neun; blue, DAPI staining of nuclei. **(B)** Western blot analysis of P-Tau under different treatment conditions and its quantification **(C)** (n = 3). **(D)** Calcein AM/PI staining images of SH-SY5Y cells under different treatment conditions and quantitative analysis of dead and live cells (n = 3). Red, PI staining of dead cell; green, Calcein AM staining of viable cells. **(E)** Representative immunofluorescence images and quantitative analysis of Aβ deposition in the hippocampal region of mice from different treatment groups (n = 3). Green, Aβ; blue, DAPI staining of nuclei. **(F)** Western blot analysis of P-Tau in mouse brain tissues from different treatment groups and its quantification **(G)** (n = 3). **(H)** Representative immunofluorescence images and quantitative analysis of P-Tau in the hippocampal region of mice from different treatment groups (n = 3). Green, P-Tau (S202/T205); blue, DAPI staining of nuclei. **(I)** Nissl staining images of the hippocampal region in mice from different treatment groups and quantitative analysis of neuronal number. The upper panels show panoramic views of the hippocampus (scale bar = 250 μm); the lower panels are magnified views of the corresponding areas indicated by black boxes (scale bar = 100 μm). The bar graph presents the statistical results of neuronal counts for the entire hippocampal region based on the panoramic images (n = 3). **(J)** Representative images of Golgi-stained mouse brain tissues from different treatment groups and quantitative analysis of dendritic spine density (n = 3). Data are presented as mean ± SD (n = 3). Statistical significance is determined by the Student's t-test based on *P* < 0.05 (*), *P* < 0.01 (**), *P* < 0.001 (***), and *P* < 0.0001 (****).

The potential cause of cognitive dysfunction in 3xTg mice could be the aggregation of pathological proteins such as Aβ and P-Tau, which were closely associated with impaired synaptic plasticity and neuronal degeneration [22]. To verify whether RVG-EXOs could promote the clearance of pathological proteins such as Aβ and P-Tau and protect neurons, the level of 647-fluorescent-labeled Aβ1-42 (647-Aβ1-42) was detected in SY5Y cells subjected to different treatments. In 24 h, cells treated with Aβ1-42 alone still exhibited a substantial amount of 647-Aβ1-42, indicating impaired intracellular Aβ clearance capacity. In contrast, treatment with either EXOs or RVG-EXOs enhanced the cells' ability to clear Aβ, with more pronounced enhancement detected after RVG-EXO treatment (Fig. 3A). These results confirmed that both EXOs and RVG-EXOs could promote the clearance of Aβ protein within cells, with RVG-EXOs exhibiting a stronger effect. To examine the effect of RVG-EXOs on Tau protein hyperphosphorylation, the level of P-Tau (at Ser396) was measured in cells treated with okadaic acid (OA) alone or followed by treatment with either EXOs or RVG-EXOs. Western blot analysis revealed that OA treatment significantly increased Tau phosphorylation at the Ser396 site compared to the normal control group. Both EXOs and RVG-EXOs reduced the level of P-Tau to some extent, with RVG-EXOs showing a stronger effect (Fig. 3B and C).

Additionally, it was observed that exposure to Aβ1-42 severely impaired neurite outgrowth. Both EXOs and RVG-EXOs partially rescued the neurite morphology, with RVG-EXO treatment demonstrating the most effective restoration of neurite length (Fig. S2A). Calcein-AM/PI staining revealed that treatment with Aβ1-42 led to a significant increase in the number of dead cells and a corresponding decrease in the number of live cells. The addition of either EXOs or RVG-EXOs provided a certain degree of neuronal protection, reducing neuronal death, with RVG-EXOs demonstrating a stronger cytoprotective effect (Fig. 3D). In summary, these results indicated that RVG-EXOs could effectively promote the clearance of pathological proteins, such as Aβ and P-Tau, and protect neurons in vitro.

Next, we further examined whether RVG-EXOs could reduce Aβ and P-Tau pathology and protect neurons in vivo. First, immunofluorescence staining of Aβ was performed on mouse brain tissues. The results showed that, compared with the Tg-Vehicle group, the Aβ plaques in the hippocampal region of the mouse brains were reduced to some extent after treatment with either RVG-EXOs or EXOs, with the more pronounced effect detected in the RVG-EXO-treated group (Fig. 3E). Subsequently, the effect of RVG-EXOs on P-Tau were investigated in mouse brain tissues. Western blot analysis of the brain tissues revealed that the level of P-Tau at the Ser396 site was upregulated in 9-month-old 3xTg mice. Both RVG-EXOs and EXOs reduced the level of P-Tau, with the more significant reduction observed in the RVG-EXOs-treated group (Fig. 3F and G). Immunofluorescence staining of P-Tau further confirmed that RVG-EXOs significantly reduced the level of P-Tau (Fig. 3H).

Synaptophysin (SYAP) is a marker protein of neuronal synapses and is widely considered a reliable indicator of synaptic plasticity and function [23]. Our results revealed a significant decrease in SYAP expression in 3xTg mice, indicating impaired synaptic plasticity and function, which was closely associated with the previously observed cognitive dysfunction. Following treatment with either RVG-EXOs or EXOs, the level of SYAP in 3xTg mice was restored, with the treatment of RVG-EXOs demonstrating a more pronounced effect (Fig. S2B). Furthermore, Nissl staining indicated that treatment with either RVG-EXOs or EXOs could suppress neuronal loss, with the more pronounced effect detected in the treatment of RVG-EXOs (Fig. 3I). Dendritic spines, serving as the primary sites for excitatory synaptic input in brain tissue, are closely associated with synaptic activity and likely play a key role in synaptic transmission and plasticity, forming the cellular basis for learning and plasticity in the brain [24]. Golgi staining revealed a significant reduction in dendritic spine density in the brain tissues of 3xTg mice. Notably, treatment with either RVG-EXOs or EXOs restored the dendritic spine density in the mouse brain tissues, with the more pronounced effect revealed in the treatment of RVG-EXOs (Fig. 3J).

### 2.4 RVG-EXOs enhance neuronal autophagy function both in vitro and in vivo

**Fig. 4:**
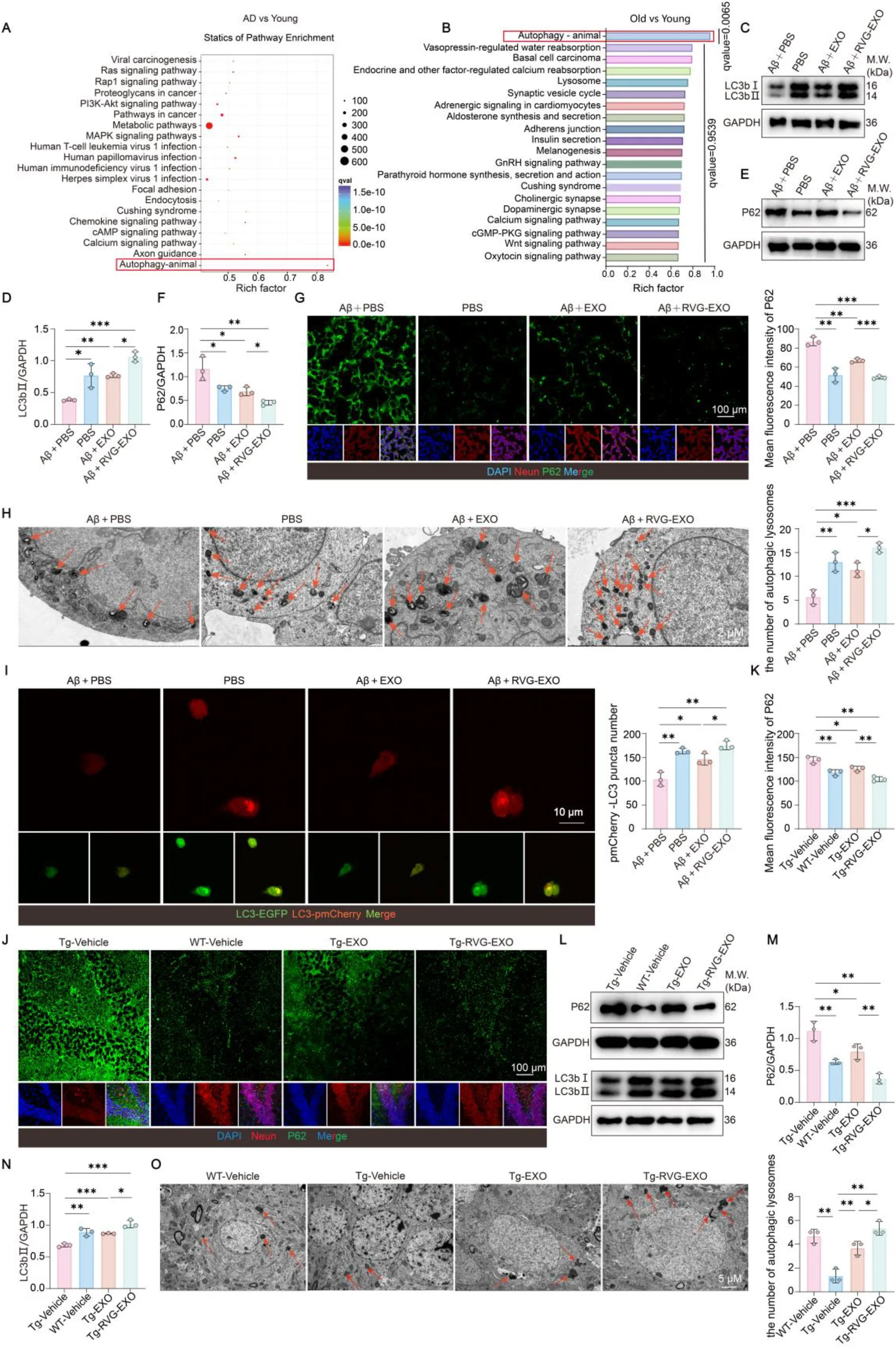
RVG-EXOs enhance neuronal autophagy function both in vitro and in vivo. MiRNA sequencing analysis is performed on plasma EXOs derived from six young individuals, six elderly individuals, and six patients with AD. **(A)** KEGG pathway enrichment analysis of target genes based on the differential miRNA expression profiles of plasma EXOs from AD patients and young individuals, revealing a significant enrichment of these target genes in the autophagy pathway. **(B)** KEGG pathway enrichment analysis of target genes based on differential miRNA expression profiles of plasma EXOs from elderly and young individuals, demonstrating significant enrichment of these target genes in the autophagy pathway. **(C)** Western blot analysis of LC3B in SH-SY5Y cells under different treatments and its quantification **(D)** (n = 3). **(E)** Western blot analysis of P62 in SH-SY5Y cells under different treatments and its quantification **(F)** (n = 3). **(G)** Representative immunofluorescence images of P62 in SH-SY5Y cells under different treatments and its quantitative analysis (n = 3). Blue, DAPI staining of nuclei; red, Neun; green, P62. **(H)** Representative TEM images of cells under different treatments and quantitative analysis of autolysosomes. Red arrows indicate autolysosomes. **(I)** Representative image and quantification of autophagic flux in SY5Y cells transfected with pmCherry-EGFP-LC3b after different treatments (n = 3). Red, pmCherry-LC3; green, EGFP-LC3. pmCherry-LC3 puncta represent autolysosomes; EGFP-LC3 puncta correspond to autophagosomes. **(J)** Representative immunofluorescence images of P62 in the hippocampal Dentate Gyrus (DG) region of mice under different treatments and its quantitative analysis **(K)** (n = 3). Blue, DAPI staining of nuclei; red, Neun; green, P62. **(L)** Western blot analysis of P62 and LC3B in mouse brain tissues under different treatments. **(M)** Quantitative analysis of P62 protein in mouse brain tissues (n = 3). **(N)** Quantitative analysis of LC3B-II protein in mouse brain tissues (n = 3). **(O)** Representative TEM images of mouse brain tissues under different treatments and quantitative analysis of autolysosomes (n = 3). Red arrows indicate autolysosomes. Data are presented as mean ± SD (n = 3). Statistical significance is determined by the Student's t-test based on *P* < 0.05 (*), *P* < 0.01 (**), and *P* < 0.001 (***).

Previous studies have demonstrated that EXOs serve as key mediators of intercellular communication, primarily through the transport of non-coding RNAs, particularly miRNAs [25,26]. Upon entering recipient cells, these miRNAs exert biological effects by influencing target gene expression via post-transcriptional regulatory networks. To investigate the mechanisms by which RVG-EXOs cleared pathological proteins such as Aβ and P-Tau and protected neurons, miRNA sequencing was performed on plasma-derived EXOs from six young individuals, six elderly individuals, and six patients with AD. KEGG pathway enrichment analysis revealed that, compared with EXOs from the elderly individuals or AD patients, the target genes of differentially expressed miRNAs in young plasma-derived EXOs were significantly enriched in autophagy-related pathways (Fig. 4A and B). Autophagy is recognized as a crucial protein degradation pathway capable of clearing misfolded proteins in various neurodegenerative diseases, including AD [7,14,15]. Additionally, the preparation of RVG-EXOs involved only conjugating the RVG29 to the exosomal membrane surface using DSPE-PEG2000, without altering their internal molecular composition. Therefore, it was speculated that the engineered young plasma-derived EXOs primarily exerted their effects by modulating the autophagy pathway. Microtubule-associated protein 1 light chain 3β (MAP1LC3B, commonly known as LC3B-I) is a widely used autophagy marker protein predominantly localized in the cytoplasm. During autophagy initiation, LC3B-I undergoes lipidation and is converted into LC3B-II, which is then recruited to the autophagosomal membrane to participate in autophagosome formation [7]. Another commonly used autophagy marker, SQSTM1/p62, is an adaptor protein that binds to LC3B and mediates the targeting of ubiquitinated protein aggregates for degradation via the autophagy pathway [27]. Aβ1-42 monomers are frequently employed to establish an AD cell model with impaired autophagy regulation in vitro [28]. Following exposure to 20 μM Aβ1-42 for 48 h, SY5Y cells exhibited decreased expression of LC3B-II and increased expression of P62, indicating suppressed autophagy in SY5Y cells [29]. In our study, after treatment with either RVG-EXOs or EXOs for 24 h, the expression level of LC3B-II was elevated, whereas the expression level of P62 was reduced, suggesting that both RVG-EXOs and EXOs partially restored the neuronal autophagy impairment induced by Aβ1-42, with RVG-EXOs demonstrating a stronger effect (Fig. 4C–F). This observation was further confirmed by immunofluorescence staining of both LC3B and P62 (Figs. S3A and 4G). TEM analysis revealed that the number of autolysosomes was significantly reduced in cells pretreated with Aβ1-42. In contrast, treatment with either RVG-EXOs or EXOs restored the number of autolysosomes, with the effect of RVG-EXOs being more pronounced (Fig. 4H). Immunofluorescence imaging demonstrated that, compared with cells treated solely with Aβ1-42, cells treated with RVG-EXOs exhibited a significant increase in the number of red puncta, representing autolysosomes (Fig. 4I). In summary, these results indicated that RVG-EXOs could enhance autophagy function in neurons in vitro.

To further validate whether RVG-EXOs could improve autophagy function in the brain tissues of 3xTg mice, immunofluorescence staining was first employed to examine the expression of the autophagic substrate P62. The results showed that after treatment of RVG-EXOs, the level of P62 protein in the brain tissues of the mice was significantly reduced (Fig. 4J and K). These findings indicated an improvement in autophagic activity in the mouse brain tissues. Subsequently, the expression of autophagy-related proteins LC3B and P62 was assessed in mouse brain tissues via Western blot analysis. The results revealed that the level of LC3B-II protein was decreased, whereas the expression level of P62 protein was increased in the brain tissues of 3xTg mice, indicating impaired autophagy function. In contrast, treatment of RVG-EXOs elevated the level of LC3B-II protein and reduced the level of P62 protein, thereby partially restoring autophagy function in the mouse brain tissues (Fig. 4L–N). TEM analysis of mouse brain tissues revealed that treatment of RVG-EXOs significantly increased the number of autolysosomes (Fig. 4O). These results indicated that RVG-EXOs could partially restore the autophagy function in the brain tissues of 3xTg mice.

### 2.5 In vitro, RVG-EXOs primarily enhance neuronal autophagy, promote the clearance of pathological proteins such as Aβ and P-Tau, and protect neurons by inhibiting the expression of the *RPTOR* gene

**Fig. 5:**
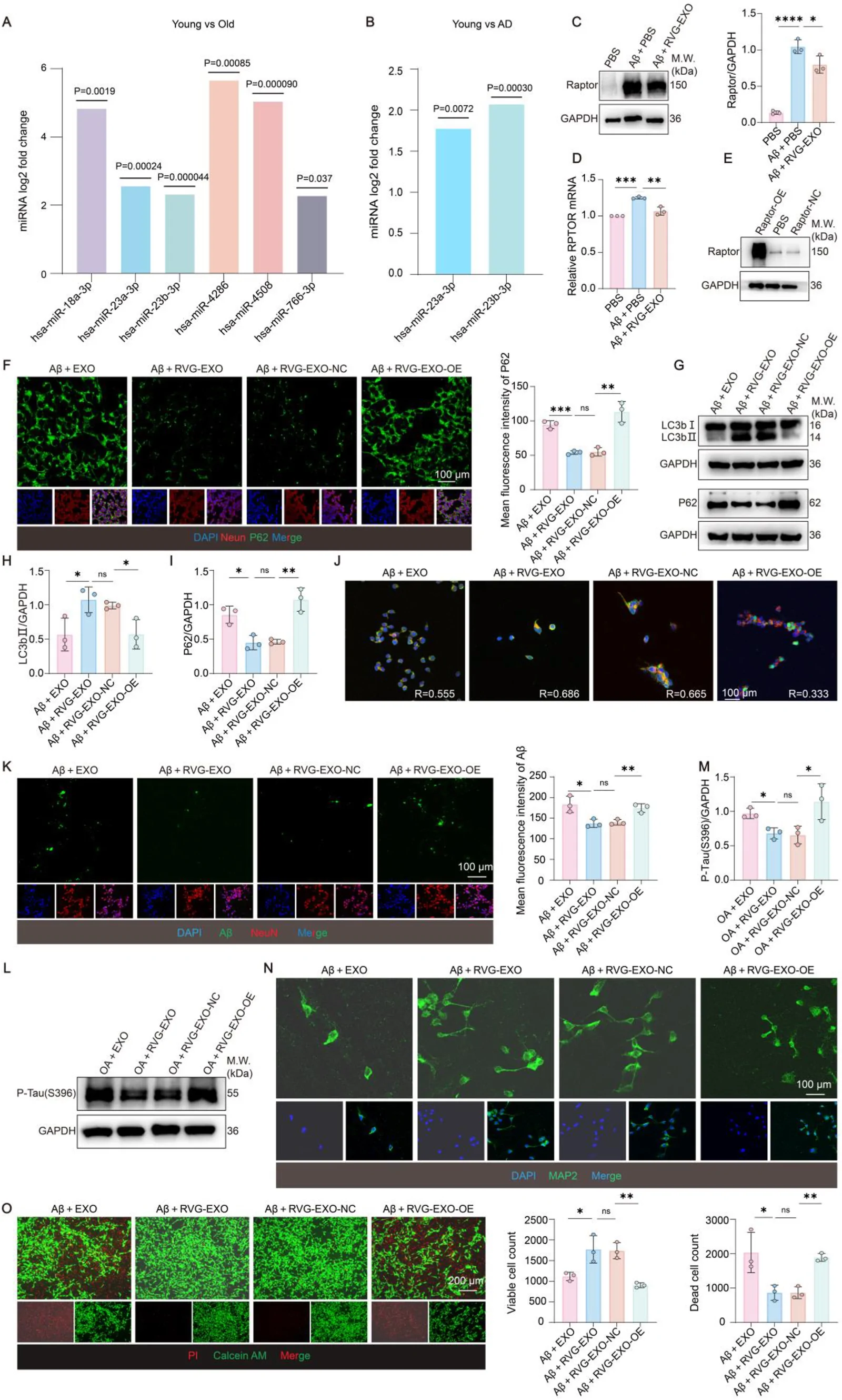
RVG-EXOs primarily enhance neuronal autophagy, promote the clearance of pathological proteins such as Aβ and P-Tau, and protect neurons by inhibiting the expression of the *RPTOR* gene in vitro. MiRNA sequencing analysis is performed on plasma EXOs derived from six young individuals, six elderly individuals, and six patients with AD. **(A)** MiRNAs significantly more abundant in plasma EXOs from young individuals compared to the elderly individuals and targeting the *RPTOR* gene. **(B)** MiRNAs significantly more abundant in plasma EXOs from young individuals compared to those of AD patients and targeting the R*PTOR* gene. **(C)** Western blot analysis and quantification of Raptor protein in cells under different treatments (n = 3). Using untreated SH-SY5Y cells as the control, the experiment shows changes in protein expression after 48-h pretreatment with 20 μM Aβ1-42 followed by 24-h co-culture with either PBS or RVG-EXOs. **(D)** Quantitative qPCR analysis showing *RPTOR* mRNA expression levels in cells under different treatments (n = 3). **(E)** Western blot analysis of Raptor protein in SH-SY5Y cells after transfection with *RPTOR* overexpression lentivirus (RPTOR-OE) or empty lentivirus (RPTOR-NC). **(F)** Immunofluorescence images and quantification of P62 protein in cells under different treatments (n = 3). Green, P62; red, Neun; blue, DAPI staining of nuclei. (**G)** Western blot analysis of LC3B and P62 proteins in cells under different treatments. **(H)** Quantification of LC3B-II protein expression levels in cells under different treatments (n = 3) based on Western blot analysis. **(I)** Quantification of P62 protein expression levels in cells under different treatments (n = 3) based on Western blot analysis. **(J)** Immunofluorescence co-localization images of LC3B and LAMP1 in cells under different treatments. Green fluorescence indicates LC3B (autophagosome marker), red fluorescence indicates LAMP1 (lysosome marker), and yellow regions show their co-localization (Merge), representing autolysosomes. The Pearson's correlation coefficient R represents the overlap ratio calculated by ImageJ (n = 3). **(K)** Representative immunofluorescence images and quantitative analysis of 647-Aβ1-42 in cells under different treatments (n = 3). Green, 647-Aβ1-42; red, Neun; blue, DAPI staining of nuclei. **(L)** Western blot analysis and quantification of P-Tau in cells **(M)** following different treatments (n = 3). **(N)** Immunofluorescence micrographs showing MAP2 in SH-SY5Y cells under different treatments. Green, MAP2; DAPI staining of nuclei. **(O)** Representative Calcein-AM/PI double-stained fluorescence images of SH-SY5Y cells and the corresponding quantification of dead and live cells following different treatments (n = 3). Red, PI staining of dead cell; green, Calcein AM staining of viable cells. Data are presented as mean ± SD (n = 3). Statistical significance is determined by the Student's t-test based on *P* < 0.05 (*), *P* < 0.01 (**), *P* < 0.001 (***), and *P* < 0.0001 (****); ns, not significant.

To further investigate the potential mechanisms by which RVG-EXOs enhanced neuronal autophagy, promoted the clearance of pathological proteins such as Aβ and P-Tau, and protected neurons, an in-depth analysis of the miRNA sequencing data was performed. The results revealed that, compared to plasma EXOs from elderly individuals, young plasma-derived EXOs exhibited significantly higher levels of hsa-miR-18a-3p, hsa-miR-23a-3p, hsa-miR-23b-3p, hsa-miR-4286, hsa-miR-4508, and hsa-miR-766-3p. Furthermore, compared to plasma EXOs from AD patients, young plasma-derived EXOs showed significantly elevated levels of hsa-miR-23a-3p and hsa-miR-23b-3p (Fig. 5A and B), and all six of these miRNAs were computationally predicted to target the *RPTOR* gene. Given that the expression levels of hsa-miR-23a-3p and hsa-miR-23b-3p were significantly upregulated in plasma EXOs from healthy young individuals compared to those from both elderly individuals and patients with AD, we further conducted target validation and functional studies on these two miRNAs. First, we examined the direct binding of hsa-miR-23a-3p and hsa-miR-23b-3p to the 3′ UTR of the *RPTOR* gene using dual-luciferase reporter assays. The results showed that co-transfection of hsa-miR-23a-3p or hsa-miR-23b-3p mimics with a wild-type reporter plasmid containing the *RPTOR*-3′ UTR led to a significant decrease in relative luciferase activity, whereas no significant change was observed when the miRNA mimics were co-transfected with a reporter plasmid carrying mutated binding sites (Fig. S4A). This indicated that hsa-miR-23a-3p and hsa-miR-23b-3p could directly target the 3′ UTR of *RPTOR*. Furthermore, we transfected SH-SY5Y cells with inhibitors of hsa-miR-23a-3p or hsa-miR-23b-3p; qRT-PCR analysis confirmed that the levels of hsa-miR-23a-3p and hsa-miR-23b-3p were significantly reduced (Fig. S4B). Subsequently, changes in endogenous Raptor protein expression were detected by Western blot. The results showed that inhibition of endogenous miR-23a-3p or miR-23b-3p led to a significant increase in Raptor protein levels in the cells (Fig. S4C and D). Taken together, these results confirmed that both hsa-miR-23a-3p and hsa-miR-23b-3p could target the *RPTOR* gene and negatively regulate its expression. Given that the preparation of RVG-EXOs involved only conjugating the RVG29 peptide to the exosomal membrane using DSPE-PEG2000 without altering their internal molecular cargo, RVG-EXOs were also expected to carry these miRNAs. To verify this, we performed qRT-PCR analysis to compare the levels of these six miRNAs between unmodified EXOs and RVG-EXOs. The results showed no significant differences in the expression levels of hsa-miR-18a-3p, hsa-miR-23a-3p, hsa-miR-23b-3p, hsa-miR-4286, hsa-miR-4508, and hsa-miR-766-3p between EXOs and RVG-EXOs (Fig. S4E–J). Furthermore, Western blot analysis demonstrated that Aβ1-42 treatment upregulated the expression of Raptor protein in SH-SY5Y cells, whereas treatment of RVG-EXOs reduced Raptor protein expression (Fig. 5C). These results suggested that RVG-EXOs could partially suppress the expression of the *RPTOR* gene, a finding further corroborated by qPCR results (Fig. 5D). Raptor, a core scaffolding protein of the mTORC1 complex, plays a central negative regulatory role in cellular autophagy by mediating the inhibitory effect of mTORC1 on the ULK1 autophagy initiation complex [30]. Therefore, it was hypothesized that RVG-EXOs likely enhanced cellular autophagic activity primarily by suppressing the expression of the *RPTOR* gene. To test this hypothesis, an *RPTOR*-overexpressing SH-SY5Y cell model was established using lentiviral transfection (Fig. 5E). Immunofluorescence results revealed that, compared with the control group (Tg-RVG-EXO-NC), *RPTOR* overexpression (Tg-RVG-EXO-OE) significantly increased the level of P62 protein and decreased the expression of LC3B-II protein in cells (Fig. 5F and S4K). Western blot analysis further confirmed this finding (Fig. 5G–I). LAMP1 and LC3B are characteristic marker proteins of the lysosomal membrane and autophagosomal membrane, respectively. Immunofluorescence colocalization analysis revealed that *RPTOR* overexpression significantly attenuated the colocalization of LAMP1 and LC3B (Fig. 5J). In summary, these results demonstrated that *RPTOR* overexpression could significantly counteract the ameliorative effects of RVG-EXOs on autophagy in SH-SY5Y cells, suggesting that RVG-EXOs enhanced neuronal autophagy primarily through the suppression of *RPTOR* gene expression.

Next, we further assessed whether RVG-EXOs exerted their effects of promoting pathological protein clearance and protecting neurons by inhibiting the expression of the *RPTOR* gene. Immunofluorescence detection revealed that under *RPTOR*-overexpressing conditions, the fluorescence intensity of 647-Aβ1-42 in the cells was significantly enhanced (Fig. 5K). Western blot analysis further demonstrated that following *RPTOR* overexpression, the expression level of P-Tau protein was markedly increased (Fig. 5L and M). Immunofluorescence staining of MAP2 revealed that *RPTOR* overexpression resulted in a significant reduction in neurite length (Fig. 5N). Additionally, Calcein-AM/PI double staining demonstrated that *RPTOR* overexpression led to a significant decrease in the number of live cells, while the number of dead cells was increased correspondingly (Fig. 5O). Collectively, these findings indicated that *RPTOR* overexpression significantly counteracted the ability of RVG-EXOs to promote the clearance of pathological proteins (such as Aβ and P-Tau) and to protect neurons. These results further suggested that RVG-EXOs exerted these beneficial effects primarily by inhibiting the expression of the *RPTOR* gene.

### 2.6 In vivo, RVG-EXOs improve brain tissue autophagy, alleviate Aβ and P-Tau pathology, and enhance cognitive behaviors in mice by suppressing the expression of the *RPTOR* gene

**Fig. 6:**
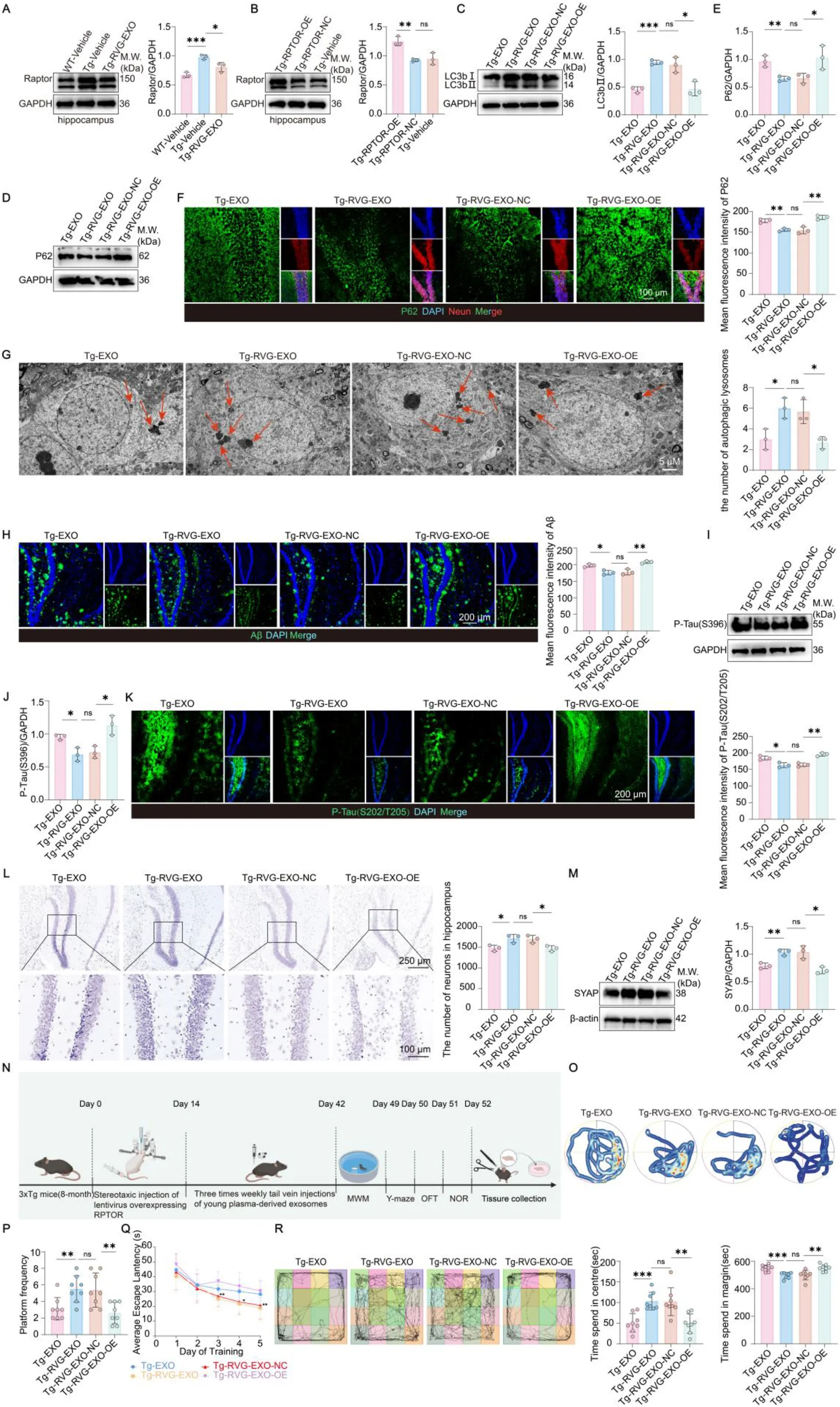
RVG-EXOs improve brain tissue autophagy, alleviate Aβ and P-Tau pathology, and enhance cognitive behaviors in mice by suppressing *RPTOR* gene expression in vivo. **(A)** Western blot analysis and quantification of Raptor protein in the hippocampal tissue of mouse brain following different treatments (n = 3). **(B)** Western blot analysis and quantification of Raptor protein in mouse hippocampal tissue (n = 3), showing the protein expression levels of Raptor in the hippocampal region of 3xTg mice following stereotaxic injection with either an *RPTOR*-overexpressing lentivirus (Tg-RPTOR-OE) or an empty lentivirus control (Tg-RPTOR-NC). **(C)** Western blot analysis and quantification of LC3B protein in mouse brain tissues after different treatments (n = 3). **(D)** Western blot analysis of P62 protein and its quantification **(E)** in mouse brain tissues under different treatments (n = 3). **(F)** Immunofluorescence images and quantitative analysis of P62 protein in hippocampal neurons of mouse brain tissues under different treatments (n = 3). Green, P62; red, Neun; blue, DAPI staining of nuclei. **(G)** Representative TEM images of brain tissues from each group of mice and quantitative analysis of autophagolysosomes (n = 3). Red arrows indicate autophagolysosome structures. **(H)** Representative immunofluorescence images and quantitative analysis of Aβ plaques in the hippocampal tissue of mice from each experimental group (n = 3). Green, Aβ; blue, DAPI staining of nuclei. **(I)** Western blot analysis of P-Tau in mouse brain tissues and its quantitative analysis **(J)** across experimental groups (n = 3). **(K)** Representative immunofluorescence images and quantitative analysis of P-Tau in the hippocampal region of mouse brain tissues across experimental groups (n = 3). Green, P-Tau (S202/T205); blue, DAPI staining of nuclei. **(L)** Nissl staining of neurons and quantitative analysis of neuron numbers in the hippocampal region across experimental groups of mice. The top panel displays a panoramic view of the hippocampus (scale bar = 250 μm), and the lower panel shows a magnified view of the corresponding area outlined in black (scale bar = 100 μm). The bar graph presents the statistical results of neuron counts for the entire hippocampal region based on the panoramic images (n = 3). **(M)** Western blot analysis of SYAP in mouse brain tissues and its quantitative analysis across experimental groups (n = 3). **(N)** Schematic diagram of the mouse behavioral experiment. **(O)** Representative trajectory heatmaps from the platform exploration test of MWM for mice in each experimental group. **(P)** Quantitative analysis of the number of target platform crossings during the MWM probe test for mice in each experimental group (n = 8). **(Q)** Representative escape latency plots for the MWM test across groups of mice (n = 8). **(R)** Representative OFT tracks and analysis of center versus periphery time (n = 8). Data are presented as mean ± SD. Statistical significance is determined by the Student's t-test based on *P* < 0.05 (*), *P* < 0.01 (**), and *P* < 0.001 (***); ns, not significant.

Upon the establishment that RVG-EXOs functioned by inhibiting *RPTOR* gene expression in vitro, animal experiments were further performed to investigate whether, in vivo, they similarly operated through the RPTOR-mediated autophagy pathway to alleviate cerebral Aβ and P-Tau pathological deposition and improve cognitive behaviors in AD model mice. Western blot analysis revealed that Raptor protein expression was significantly upregulated in the hippocampal tissues of 3xTg mice compared with wild-type mice, whereas its expression level was markedly downregulated following RVG-EXOs intervention (Fig. 6A). Western blot analysis of cortical tissues also exhibited a comparable trend (Fig. S5A). These results indicated that RVG-EXOs could also downregulate *RPTOR* expression in vivo. To further verify whether RVG-EXOs exerted their effects by suppressing *RPTOR* gene expression in vivo, an *RPTOR*-overexpression model was established in the mouse brain via stereotactic injection of an *RPTOR*-overexpressing lentivirus. Subsequently, the upregulation of Raptor protein in this model was confirmed by Western blot analysis (Fig. 6B and S5B). Thereafter, the experimental mice were subjected to grouped interventions and behavioral tests, and brain tissues were collected at the experimental endpoint for subsequent analysis. If the in vivo effects of RVG-EXOs similarly depended on the suppression of *RPTOR* expression, then *RPTOR* overexpression should significantly attenuate their therapeutic effects. Western blot results demonstrated that, compared with the control group (Tg-RVG-EXO-NC), the *RPTOR*-overexpressing group (Tg-RVG-EXO-OE) exhibited a significant decrease in the level of LC3B-II protein and a marked increase in P62 protein expression in mouse brain tissues (Fig. 6C–E). Immunofluorescence analysis further confirmed that under *RPTOR*-overexpressing conditions, the expression level of P62 protein in brain tissues was significantly upregulated (Fig. 6F). These results indicated that *RPTOR* overexpression significantly counteracted the beneficial effects of RVG-EXOs on autophagy in mouse brain tissues. TEM analysis revealed that under *RPTOR*-overexpressing conditions, the number of autolysosomes in mouse brain tissues was significantly reduced (Fig. 6G). In summary, these results demonstrated that overexpression of the *RPTOR* gene significantly inhibited the improvement of autophagic function in mouse brain tissues mediated by RVG-EXOs, indicating that RVG-EXOs primarily enhanced brain tissue autophagy by suppressing the expression of the *RPTOR* gene.

We further investigated whether RVG-EXOs alleviated Aβ and P-Tau protein pathology, protected neurons, and exerted their therapeutic effects in vivo by inhibiting the expression of the *RPTOR* gene. Immunofluorescence analysis of mouse brain tissues revealed that, compared with the control group (Tg-RVG-EXO-NC), *RPTOR* overexpression resulted in a significant increase in the number of Aβ plaques in the brain (Fig. 6H). Western blot analysis of P-Tau protein demonstrated that under conditions of *RPTOR* overexpression, the level of P-Tau protein in mouse brain tissues was significantly elevated (Fig. 6I and J). Immunofluorescence analysis of P-Tau protein further corroborated this finding (Fig. 6K). Nissl staining of the mouse hippocampal region revealed that *RPTOR* overexpression exacerbated the loss of hippocampal neurons (Fig. 6L). Western blot analysis further demonstrated that under conditions of *RPTOR* overexpression, the level of SYAP in mouse brain tissues was significantly decreased (Fig. 6M), indicating that *RPTOR* overexpression exacerbated hippocampal neuronal damage and markedly counteracted the neuroprotective effects of RVG-EXOs. Collectively, these results demonstrated that *RPTOR* overexpression significantly counteracted the ability of RVG-EXOs to reduce pathological protein deposition and protect neurons in brain tissues, suggesting that RVG-EXOs exerted these therapeutic effects primarily by suppressing the expression of the *RPTOR* gene.

We further explored how RVG-EXOs improved cognitive function in mice in vivo and examined whether this effect was similarly dependent on the suppression of *RPTOR* gene expression using 8-month-old 3xTg mice. An *RPTOR*-overexpression model was established in the mouse brain via stereotaxic injection of an *RPTOR*-overexpressing lentivirus; mice injected with an empty lentivirus served as controls. Subsequently, the experimental mice were subjected to grouped interventions. Upon completion of the interventions, all mice underwent a series of behavioral tests, including MWM, Y-maze, OFT, and NOR test (Fig. 6N).

First, the MWM was used to assess whether the improvement of spatial learning and memory in mice by RVG-EXOs was dependent on the suppression of the *RPTOR* gene expression. On the first day, the mice underwent visual training to assess their vision and swimming ability, followed by 5 d of hidden platform training for all groups. On the 7th day, the platform was removed, and a probe test was conducted using animal tracking software to evaluate the memory function of the mice. Notably, during the hidden platform training, compared with the empty-vector control mice (Tg-RVG-EXO-NC), the *RPTOR*-overexpressing mice exhibited slower learning ability and longer escape latencies, indicating that *RPTOR* overexpression attenuated the improvement of spatial learning ability in 3xTg mice conferred by RVG-EXOs. In the subsequent probe test, the *RPTOR*-overexpressing mice crossed the target platform significantly fewer times than the empty-vector control mice. In summary, the MWM results demonstrated that *RPTOR* overexpression significantly counteracted the improvement of spatial learning and memory in 3xTg mice by RVG-EXOs, suggesting that the cognitive-enhancing mechanisms of RVG-EXOs operated primarily through the suppression of *RPTOR* gene expression (Fig. 6O–Q).

Next, the Y-maze test was employed to assess short-term spatial working memory. The results showed that under conditions of *RPTOR* gene overexpression, the spontaneous alternation rate of the mice was significantly decreased (Fig. S5C). These results indicated that *RPTOR* overexpression could significantly counteract the improvement effect of RVG-EXOs on short-term spatial working memory, further suggesting that this improvement could be primarily achieved through the inhibition of *RPTOR* gene expression.

Subsequently, an OFT was conducted to evaluate anxiety-like behavior in the mice. Each mouse was placed in the center of a novel open arena and allowed to explore freely for 5 min. Its movement trajectory as well as the time spent in the central and peripheral zones were recorded. Notably, compared with the empty-vector control mice, the *RPTOR*-overexpressing mice showed a significant reduction in the time spent in the central zone (Fig. 6R). This indicated that *RPTOR* overexpression significantly attenuated the ameliorative effect of RVG-EXOs on anxiety-like behavior in mice, suggesting that RVG-EXOs alleviated such behavior primarily by suppressing the expression of the *RPTOR* gene.

Finally, the NOR test was conducted to assess object recognition memory in mice. The results showed that, compared with the empty-vector control mice, the *RPTOR*-overexpressing mice exhibited a significant decrease in the RI (Fig. S5D). This indicated that *RPTOR* overexpression significantly attenuated the ameliorative effect of RVG-EXOs on object recognition memory in mice, suggesting that RVG-EXOs exerted the beneficial effects primarily by inhibiting the expression of the *RPTOR* gene. In summary, these behavioral tests indicated that *RPTOR* overexpression could significantly attenuate the beneficial effects of RVG-EXOs on cognitive behaviors in mice, suggesting that RVG-EXOs improved cognitive performance primarily by inhibiting the expression of the *RPTOR* gene.

### 2.7 Single-cell RNA Sequencing Reveals that RVG-EXOs exert neuroprotection via Remodeling Neuronal Transcriptome, subtype composition, and intercellular communication networks

**Fig. 7:**
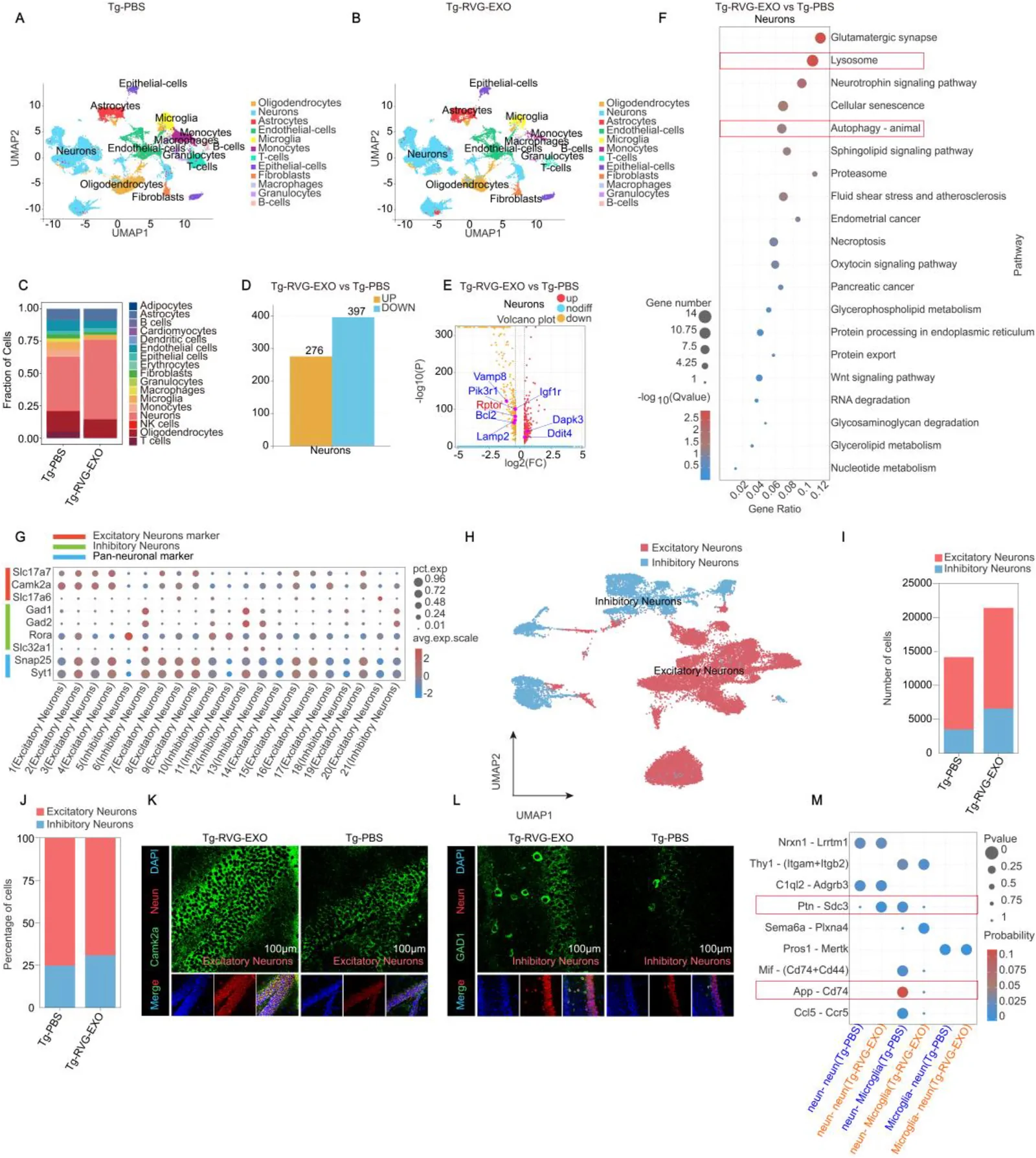
Single-Cell RNA Sequencing Reveals that RVG-EXOs Exert Neuroprotection via Remodeling Neuronal Transcriptome, Subtype Composition, and Intercellular Communication Networks. **(A)** UMAP plot showing the classification of cell subpopulations in brain tissue of PBS-treated mice. **(B)** UMAP plot showing the classification of cell subpopulations in brain tissue of RVG-EXO-treated mice. **(C)** Quantitative plot showing the proportions of different cell types in the brain tissue of PBS-treated and RVG-EXO-treated mice based on single-cell sequencing analysis. **(D)** Quantitative plot showing differentially expressed genes in neurons of brain tissue from RVG-EXO-treated 3xTg mice compared with PBS-treated controls, based on single-cell sequencing analysis. **(E)** Volcano plot showing up- and down-regulated differentially expressed genes in neurons of brain tissue from RVG-EXO-treated 3xTg mice compared with PBS-treated controls, based on single-cell sequencing analysis, with autophagy-related genes marked in the plot. **(F)** Bubble plot showing the significance of KEGG pathway enrichment for differentially expressed genes in neurons between PBS-treated and RVG-EXO-treated mice, based on single-cell sequencing analysis. **(G)** Bubble plot showing the expression of marker genes in neuronal subpopulations. The size of each bubble (pct.exp) represents the proportion of cells expressing the gene within that subpopulation, and the color (avg.exp.scale) indicates the average expression level of the gene. **(H)** UMAP plot of excitatory and inhibitory neurons. **(I)** Stacked bar plot showing the numbers of excitatory and inhibitory neurons in each treatment group. **(J)** Stacked bar chart showing the proportions of excitatory and inhibitory neurons in each treatment group. **(K)** Representative immunofluorescence images of Camk2a protein in different treatment groups. Camk2a + indicate excitatory neurons. Green, Camk2a; red, Neun; blue, DAPI staining of nuclei. **(L)** Representative immunofluorescence images of GAD1 protein in different treatment groups. GAD1+ indicate inhibitory neurons. Green, GAD1; red, Neun; blue, DAPI staining of nuclei. **(M)** Bubble plot showing differences in ligand-receptor pair expression levels in cell communication between neurons and microglia. Bubble color represents interaction probability, and bubble size represents the P-value for significance.

To further elucidate the pharmacological mechanisms of RVG-EXOs at the cellular and molecular levels, we performed single-cell RNA sequencing (scRNA-seq) on brain tissues from 3×Tg mice treated with either PBS (Tg-Vehicle) or RVG-EXOs (Tg-RVG-EXO). Unbiased clustering of all captured cells identified major cell types including Oligodendrocytes, Neurons, Astrocytes, Endothelial cells, Microglia, Monocytes, T cells, Epithelial cells, Fibroblasts, Macrophages, Granulocytes and B cells (Figs. S6A, S7A, S8A). Remarkably, compared with the Tg-Vehicle group, RVG-EXOs treatment led to a significant increase in the proportion of neurons (Fig. 7A–C). This observation is consistent with the neuroprotective effects of RVG-EXOs demonstrated by Nissl staining and dendritic spine analysis. Differential gene expression analysis of neurons revealed that, compared with the PBS control group, RVG-EXOs treatment led to significant upregulation of 276 genes and downregulation of 397 genes in neurons, including *Rptor* (Fig. 7D and E). KEGG pathway enrichment analysis of the differentially expressed genes in neurons revealed that they were significantly enriched in multiple pathways, including autophagy, lysosomal function, the neurotrophin signaling pathway, and others (Fig. 7F). Notably, the enrichment of autophagy-related genes and the downregulation of the *Rptor* gene in neurons further corroborate our mechanistic finding that RVG-EXOs promote autophagic flux in neurons by inhibiting the expression of *RPTOR*.

Given the significant increase in neuronal proportion and the critical role of different neuronal subtypes in cognitive function, we further classified neurons into excitatory (E) and inhibitory (I) subpopulations based on the expression of canonical marker genes (Excitatory: Slc17a7, Camk2a, Slc17a6 [31]; Inhibitory: Gad1, Gad2, Slc32a1, Rora [32,33]) (Fig. 7G and H). This analysis revealed that RVG-EXOs treatment not only increased the absolute number of both excitatory and inhibitory neurons but also elevated the percentage of inhibitory neurons within the total neuronal population (Fig. 7I and J). Camk2a and GAD1 serve as well-established markers for excitatory and inhibitory neurons, respectively [34]. Therefore, we performed immunofluorescence staining to examine their expression levels, thereby assessing the alterations in these two neuronal types. Immunofluorescence results also showed that RVG-EXOs treatment significantly increased the levels of excitatory and inhibitory neurons (Fig. 7K, L and S9A, B). Given that E/I imbalance is a hallmark of AD pathophysiology and its restoration is associated with cognitive improvement and protection against excitotoxicity [35], this shift in neuronal subtype composition suggests that RVG-EXOs may rebalance neural network activity in brain tissue.

To gain further mechanistic insights at the intercellular level, we performed cell-cell communication analysis, which revealed that RVG-EXOs treatment significantly strengthened the interaction between neurons via the Ptn-Sdc3 ligand-receptor pair, a pathway critically involved in promoting synaptic plasticity, neurite outgrowth, and neuronal survival [36,37]. Conversely, RVG-EXOs significantly weakened the communication between neurons and microglia through the App-Cd74 ligand-receptor pair, which is closely associated with Disease-Associated Microglia (DAM) generation and neuroinflammation (Fig. 7M). [38,39]. These findings suggest that RVG-EXOs not only intrinsically enhance neuronal health but also reshape the brain's intercellular signaling network to foster a more neuroprotective and anti-inflammatory microenvironment.

### 2.8 Single-Cell Sequencing Reveals that RVG-EXOs Suppress Pathogenic DAM Generation and Reshape Microglial Homeostasis via Autophagy-Dependent Downregulation of APP-CD74 signaling

**Fig. 8:**
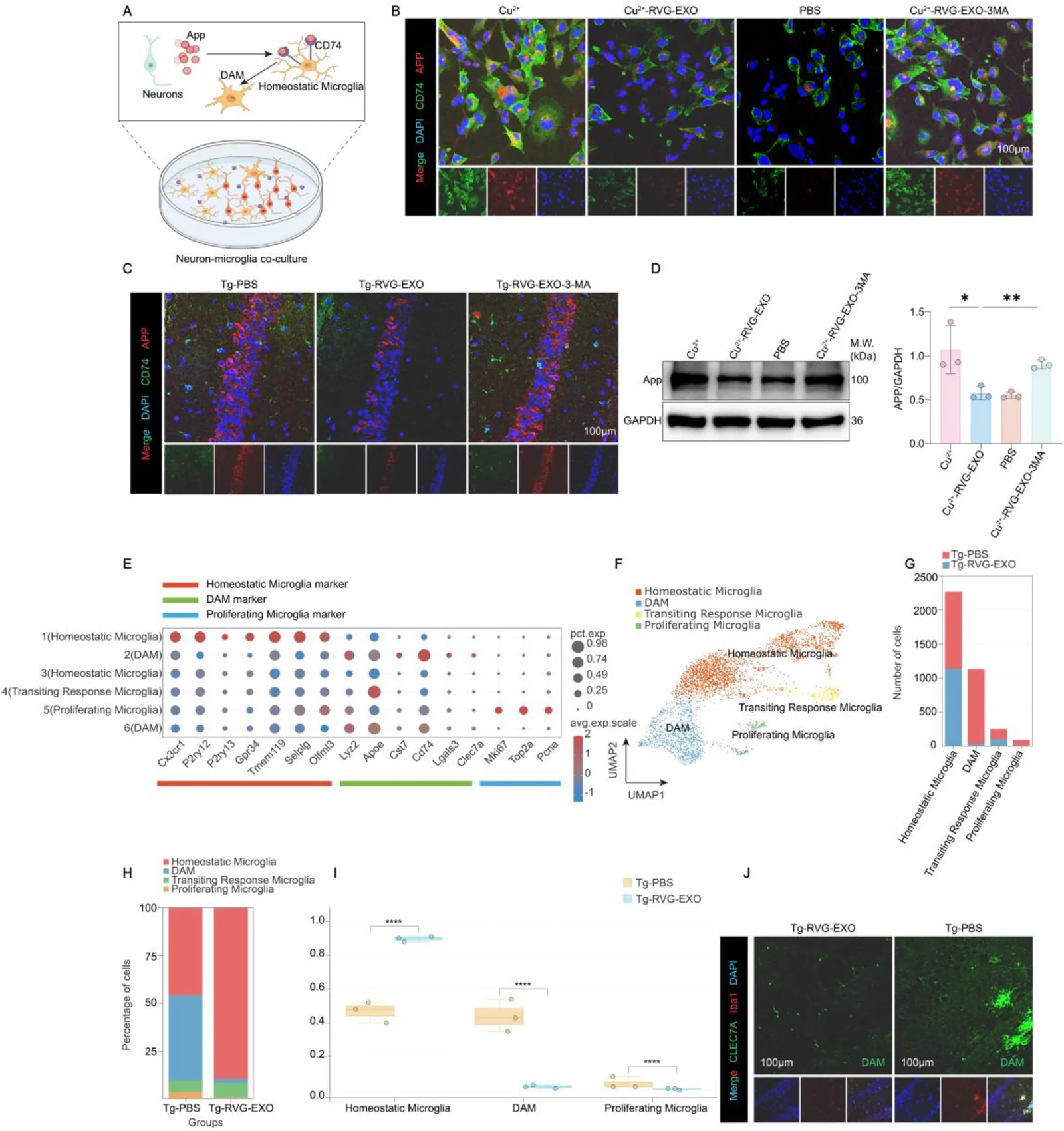
Single-Cell Sequencing Reveals that RVG-EXOs Suppress Pathogenic DAM Generation and Reshape Microglial Homeostasis via Autophagy-Dependent Downregulation of APP-CD74 Signaling. **(A)** Schematic of neuron–microglia co-culture. **(B)** Representative Immunofluorescence images of APP and CD74 proteins in cells subjected to different treatments. Green, CD74; red, APP; blue, DAPI staining of nuclei. **(C)** Representative immunofluorescence images of APP and CD74 proteins in mouse brain tissues after different treatments. Green, CD74; red, APP; blue, DAPI staining of nuclei. (**D)** Western blot analysis and quantification of APP protein in cells under various treatments (n = 3). Data are presented as mean ± SD. Statistical significance is determined by the Student's t-test based on *P* < 0.05 (*), *P* < 0.01 (**), and *P* < 0.001 (***); ns, not significant. **(E)** Bubble plot showing marker genes expression in microglial subpopulations. Bubble size (pct.exp) represents the proportion of cells expressing the gene within that subpopulation, and bubble color (avg.exp.scale) indicates the average expression level. **(F)** UMAP plot of microglial subpopulations. **(G)** Stacked bar plot showing the cell numbers of each microglial subpopulation in mouse brain tissues under different treatments. **(H)** Stacked bar plot showing the proportions of each microglial subpopulation in mouse brain tissues under different treatments. **(I)** Box plot showing differences in the proportions of microglial subpopulations in mouse brain tissues under different treatments (n = 3). Data are presented as proportions. Statistical significance was determined using the chi-square test, with P < 0.05 (*), P < 0.01 (**), P < 0.001 (***),p < 0.0001 (****); ns, not significant. **(J)** Immunofluorescence images of CLEC7A protein in mouse brain tissues from different treatment groups. CLEC7A + indicate DAM. Green, CLEC7A; red, Iba1; blue, DAPI staining of nuclei.

As mentioned above, intercellular communication analysis revealed that RVG-EXOs significantly attenuate the interaction of the App–Cd74 ligand–receptor pair between neurons and microglia, which is closely associated with DAM generation and neuroinflammation [38,39]. Immunofluorescence results of brain tissues and cells showed that RVG-EXOs treatment significantly reduced APP protein levels and diminished APP–CD74 receptor binding. However, this effect was markedly attenuated upon the addition of an autophagy inhibitor, a finding further corroborated by Western blot analysis (Fig. 8A–D and S9C-F). Collectively, these data suggest that the observed reduction in APP–CD74 ligand–receptor interactions between neurons and microglia following RVG-EXOs treatment is primarily attributable to enhanced neuronal autophagy, which lowers APP protein abundance. Considering the pivotal role of microglia in AD pathology, we next performed a sub-clustering analysis of microglial populations. Based on established markers, we identified four distinct microglial subtypes: Homeostatic Microglia (expressing Cx3cr1, P2ry12, P2ry13, Gpr34, Tmem119, Selplg, Olfml3 [40].), DAM (expressing Lyz2, ApoE, CST7, Cd74, Lgals3, Clec7a [[40], [41], [42]].), Proliferating Microglia (expressing Mki67, Top2a, Pcna [43,44]), and Transiting Response Microglia (a transitional microglial state between DAM and homeostatic microglia and ApoE levels were notably higher [45]) (Fig. 8E and F). Strikingly, RVG-EXOs treatment led to a significant decrease in both the absolute numbers and relative proportions of the deleterious DAM and Proliferating microglia subtypes, while concurrently increasing the proportion of the homeostatic, surveillant microglia (Fig. 8G–I). CLEC7A is a commonly used marker of DAM [46]. In this study, immunofluorescence staining was performed to detect its expression level, so as to reflect the changes in DAM. Immunofluorescence also revealed that treatment with RVG-EXOs significantly reduced the levels of DAM (Fig. 8J and S9G). These findings indicate that RVG-EXOs may exert neuroprotective functions by reducing the interaction between neuronal APP and microglial CD74, thereby inhibiting the generation of DAM, a phenotype closely associated with neuroinflammation [47].

Together, these single-cell transcriptomic data provide unbiased evidence that RVG-EXOs reshape brain cellular composition, promote a homeostatic microglial state, and modulate key intercellular communication networks. More importantly, integration of these findings with our mechanistic studies supports a coherent model in which RVG-EXOs target neurons and suppress *Rptor* expression, thereby activating neuronal autophagy. Enhanced autophagy directly facilitates the clearance of neurotoxic proteins, including Aβ and P-Tau, thus alleviating primary neuronal injury. In parallel, autophagy activation accelerates APP degradation and turnover, reducing the levels of APP-related ligands that are either displayed on the neuronal surface or secreted and capable of binding to microglial CD74. Consequently, neuron-to-microglia APP-CD74 signaling is attenuated, which inhibits the transition of microglia toward a pro-inflammatory DAM phenotype and thereby limits secondary neuroinflammatory damage to neurons. Through this dual mechanism—direct elimination of pathological proteins and indirect remodeling of the neuroimmune microenvironment, RVG-EXOs achieve robust neuroprotection in the Alzheimer's disease model.

### 2.9 Biosafety evaluation of RVG-EXOs and EXOs in vivo

To evaluate the long-term safety of RVG-EXOs and EXOs, we administered EXOs or RVG-EXOs to C57 mice via tail vein injection every other day for 40 d. At the end of the treatment, plasma levels of the pro-inflammatory cytokines IL-6, TNF-α, and IL-1β were measured by ELISA. No significant differences were observed among the PBS, EXO, and RVG-EXO groups, indicating that neither EXOs nor RVG-EXOs treatment elicited a systemic inflammatory response (Fig. S10A–C). Additionally, to evaluate potential immune responses, we also measured plasma levels of IgG, which showed no significant difference among the PBS, EXO, and RVG-EXO groups (Fig. S10D), suggesting that repeated administration of EXOs or RVG-EXOs caused no humoral immune response. Furthermore, major organs including the heart, liver, spleen, lung, kidney, and brain were collected for histopathological analysis. H&E staining revealed no apparent tissue damage or inflammatory cell infiltration in any of the organs examined across all treatment groups (Fig. S10E). To further assess potential local inflammation, we performed ELISA to measure the protein expression levels of the inflammatory cytokines IL-6, TNF-α, and IL-1β in tissue lysates from the liver and brain where EXOs and RVG-EXOs were predominantly distributed. Consistent with the histological findings, no significant upregulation of these inflammatory markers was detected in the EXO- or RVG-EXO-treated groups compared to the PBS control group (Fig. S10F–K). Collectively, these results demonstrate that repeated administration of EXOs or RVG-EXOs caused no detectable systemic or organ-specific inflammatory responses, immune activation, or pathological damage, supporting the biosafety of our engineered EXOs for potential therapeutic applications.

## 3 Discussion

**Fig. 9:**
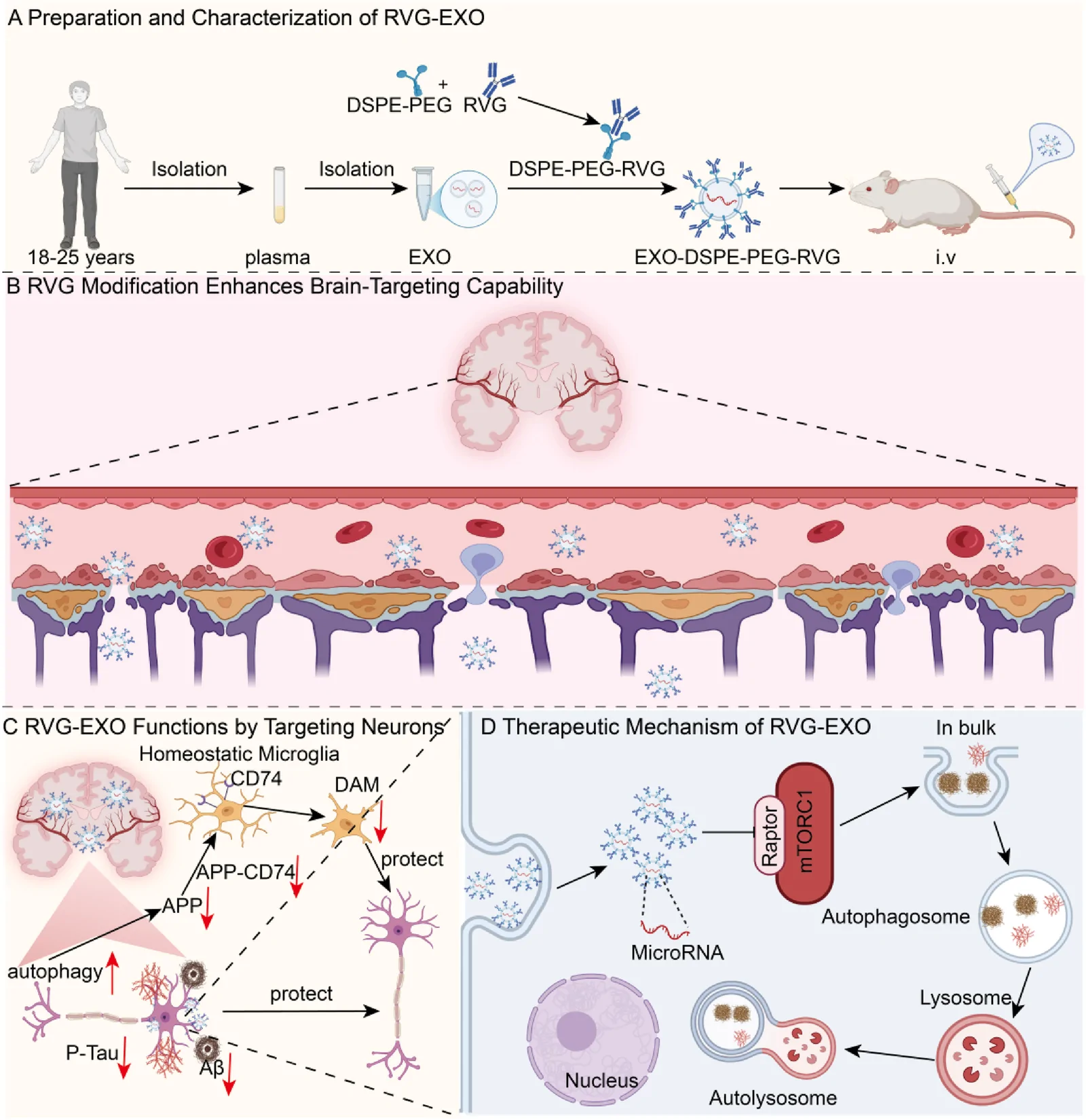
Schematic illustration of RVG-EXO fabrication and its therapeutic mechanism in the AD brain. **(A)** Schematic diagram of the RVG-EXO production. **(B)** RVG engineering enhances the brain-targeting efficiency of EXOs derived from young donors. **(C)**RVG-EXOs are capable of targeting neurons and enhancing neuronal autophagy. On one hand, this promotes the clearance of pathological proteins such as Aβ and P-Tau, thereby reducing neuronal damage. On the other hand, autophagy-mediated clearance of APP reduces the binding of APP to CD74 receptors on microglia, which in turn inhibits the microglial transition to the DAM phenotype and alleviates neuroinflammation. Through these dual pathways, RVG-EXOs collectively exert neuroprotective effects. **(D)** After entering neurons, RVG-EXOs improve autophagy and promote the clearance of pathological proteins by inhibiting Raptor expression.

This study successfully constructed RVG-EXOs and systematically elucidated their therapeutic effects and mechanisms in an AD model. Our results demonstrated that RVG-EXOs exhibited typical exosomal characteristics, and RVG modification significantly enhanced the neuronal targeting capability and brain delivery efficiency. In 3×Tg AD model mice, treatment with RVG-EXOs effectively improved multiple cognitive behavioral deficits, including spatial learning, working memory, and object recognition. At the pathological level, RVG-EXOs demonstrated strong abilities both in vitro and in vivo to promote Aβ clearance, inhibit Tau hyperphosphorylation, and protect synaptic structure and neuronal survival. Mechanistically, our study revealed that RVG-EXOs activated the autophagy pathway via targeted inhibition of RPTOR. On one hand, the enhancement of neuronal autophagy promotes the clearance of pathological proteins such as Aβ and P-Tau, thereby reducing neuronal damage. On the other hand, autophagy-mediated clearance of APP reduces the binding of APP to CD74 receptors on microglia, which in turn inhibits the microglial transition to the DAM phenotype and alleviates neuroinflammation. Through these dual pathways, RVG-EXOs collectively exert neuroprotective effects. Key rescue experiments confirmed that *RPTOR* overexpression significantly inhibited the therapeutic effects of RVG-EXOs, establishing the core functional axis of “RVG-EXO–*RPTOR*–autophagy” (Fig. 9A–D).

RVG-29 has been widely used to enhance the brain-targeted delivery efficiency of drug carriers due to its ability to specifically recognize acetylcholine receptors on the neuronal surface [48]. Studies have shown that modifying mesenchymal stem cells with RVG can significantly enhance their migration and enrichment capacity toward brain tissues [49]. Additionally, RVG-modified nanoplatelets can also effectively enhance the delivery of therapeutic agents to the brain in glioma models, further highlighting the value of the RVG peptide in overcoming the BBB and achieving central nervous system (CNS) targeting [50]. Our study successfully constructed RVG-EXOs by conjugating RVG-29 to EXOs isolated from young human plasma using a lipid anchoring method (Fig. 1A–F) and systematically evaluated its delivery efficiency. The results demonstrated that, compared to unmodified natural EXOs, RVG-EXOs exhibited a significantly enhanced cellular uptake capability when co-cultured with SH-SY5Y cells in vitro (Fig. 1G–K). In vivo experiments further confirmed that after tail vein injection, the accumulation signals of RVG-EXOs in mouse brain tissues were significantly higher than those of plain EXOs, demonstrating its enhanced BBB penetration and targeted enrichment capability in the brain (Fig. 1L and M). These results align with the previously observed trend of RVG modification in other delivery systems, validating the applicability and effectiveness of this targeting strategy in EXO engineering.

In recent years, circulating factors (such as plasma and EXOs) from young individuals have attracted significant attention for their roles in reversing age-related functional decline and ameliorating neurodegenerative diseases. Studies have shown that young plasma can reverse cognitive impairment in aged 3×Tg-AD mice [51]. Furthermore, studies have found that EXOs derived from young plasma can reverse age-related cognitive decline in aged mice by improving mitochondrial energy metabolism [9]. EXOs derived from young serum have also been proven to ameliorate age-related cognitive decline in aged mice [10]. In our study, we validated the direct therapeutic effect of RVG-EXOs in the 3×Tg AD model. Behavioral test results demonstrated that treatment of RVG-EXOs significantly improved spatial learning and memory capabilities of AD mice in the MWM, increased the spontaneous alternation rate in the Y-maze, and restored their exploration preference in the NOR test (Fig. 2A–F). These results align with the overall trend observed for “rejuvenating” factors in improving cognitive function and directly demonstrate, within an AD transgenic model, the effectiveness of engineered young plasma EXOs in alleviating multiple cognitive deficits.

The core pathological hallmarks of AD include the deposition of Aβ plaques in the brain and the formation of neurofibrillary tangles composed of P-Tau, which collectively contribute to synaptic dysfunction and neuronal death [2]. Research has shown that young plasma can reduce neuroinflammation, decrease Aβ deposition, lower the level of Tau protein hyperphosphorylation, and protect neurons [51]. Building upon this foundation, our study further constructed and validated the role of RVG-EXOs in intervening in key pathological processes of AD. In the cell model, treatment of RVG-EXOs significantly enhanced the ability of SH-SY5Y cells to clear Aβ, reduced the levels of P-Tau induced by OA, and effectively alleviated neurite damage and cell death caused by Aβ exposure (Fig. 3A–D and S2A, B). In 3×Tg AD model mice, treatment of RVG-EXOs likewise demonstrated clear therapeutic effects: it significantly reduced Aβ plaque burden in the hippocampus, decreased P-Tau protein expression, restored SYAP expression and dendritic spine density, and improved neuronal survival rates (Fig. 3E–J). These results indicate that RVG-EXOs not only retain the neuroprotective activity of young plasma-derived EXOs but also, through enhanced targeted delivery efficiency, effectively modulate the core pathological processes of AD in both in vitro and in vivo systems.

RPTOR serves as the core scaffold protein of the mTORC1 complex. By inhibiting the activity of the ULK1/2 autophagy initiation complex, it negatively regulates the cellular autophagy process [52]. Autophagy is recognized as a key protein degradation pathway responsible for clearing misfolded proteins and plays a key role in various neurodegenerative diseases, including AD [7,14,15]. Studies indicate that autophagic dysfunction is an early and core pathological feature of AD, forming a vicious cycle with the accumulation of Aβ and P-Tau [53]. Therefore, restoring neuronal autophagic function has been regarded as a crucial strategy for intervening in AD, which can significantly mitigate pathological protein deposition, reduce neuronal loss, and improve cognitive behavior [54]. The most critical mechanistic finding of this study lies in elucidating the complete pathway through which young plasma-derived EXOs and RVG-EXOs exert neuroprotective effects by targeting the RPTOR-mTORC1 axis to activate autophagy. We first confirmed that both AD cell and animal models exhibited impaired autophagic flux, as indicated by abnormal LC3B-II alterations and P62 accumulation. Treatment with young plasma-derived EXOs or RVG-EXOs effectively reversed this phenomenon (Fig. 4C–G, 4J–N, and S3A). Further mechanistic investigation traces the origin to RPTOR, a core component of mTORC1. Studies suggest that *RPTOR* is a susceptibility gene for AD and may represent a potential therapeutic target [55]. Based on sequencing and bioinformatics analyses, a total of six miRNAs, e.g., hsa-miR-23a-3p and hsa-miR-23b-3p, were identified and highly expressed in plasma EXOs and RVG-EXOs from young adults and could target *RPTOR* gene (Fig. 5A and B). Subsequent in vitro and in vivo experiments confirmed that RVG-EXOs could downregulate *RPTOR* expression. Conversely, artificial overexpression of *RPTOR* significantly inhibited the beneficial effects of RVG-EXOs across multiple levels, including improving autophagy, clearing pathological proteins, protecting neurons, and ameliorating behavioral outcomes (Fig. 5C–O, 6A–R, S4K, and S5A–D). This series of evidence collectively and conclusively demonstrates the following causal functioning flow: plasma EXOs and RVG-EXOs from young individuals → inhibition of *RPTOR* → relief of mTORC1-mediated suppression of autophagy → restoration of autophagic flux → clearance of pathological proteins and neuroprotection. The elucidation of this mechanism elevates the understanding of young plasma EXOs in treating AD from a phenotypic description to the level of a clearly defined molecular pathway.

Our single-cell transcriptomic profiling further extended these mechanistic insights by revealing the cellular and microenvironmental remodeling induced by RVG-EXOs in the 3×Tg mouse brain. Consistent with the neuroprotective effects observed histologically, RVG-EXO treatment significantly increased the proportion of neurons (Fig. 7A–C). More importantly, the downregulation of *Rptor* expression in neurons and the significant enrichment of autophagy pathways at the single-cell level provided direct in vivo evidence supporting our central mechanistic axis (Fig. 7D–F). This finding corroborates our biochemical and functional data, reinforcing the conclusion that RVG-EXOs promote autophagic flux in neurons through the RPTOR-mTORC1 axis. In addition, studies have found that excitation/inhibition (E/I) imbalance is a hallmark of Alzheimer's disease pathophysiology, and that restoring this balance is associated with improved cognitive function and protection against excitotoxicity [35]. Our single-cell sequencing results show that RVG-EXOs not only increase the absolute numbers of both excitatory and inhibitory neurons, but also elevate the proportion of inhibitory neurons. This alteration contributes to restoring E/I balance and ameliorating cognitive deficits in AD (Fig. 7I and J).

Beyond neuronal changes, the single-cell data unveiled a previously unrecognized effect of RVG-EXOs on microglial dynamics. We observed a significant reduction in the deleterious DAM and proliferating microglia subsets, alongside a concurrent increase in the homeostatic microglial population (Fig. 8G–J). Given that DAM are known to perpetuate neuroinflammation and contribute to synaptic loss in AD [47], this microglial repolarization toward a homeostatic phenotype represents a critical secondary mechanism by which RVG-EXOs exert neuroprotection. This is further supported by the cell-cell communication analysis, which revealed that RVG-EXOs strengthened the neurotrophic Ptn-Sdc3 signaling between neurons while attenuating the App-CD74 signaling axes between neurons and microglia that promotes DAM generation. (Fig. 7M). Collectively, these findings suggest a dual-action model for RVG-EXOs: they directly activate neuronal autophagy to clear pathological proteins and, in parallel, remodel the brain's intercellular communication network to foster an anti-inflammatory, neurosupportive microenvironment. This integrated perspective, derived from unbiased single-cell analysis, not only validates our proposed RPTOR-autophagy mechanism but also uncovers a broader therapeutic landscape wherein RVG-EXOs harmonize both neuronal and glial functions to combat AD pathology.

Several studies have explored RVG-modified EXOs for AD therapy. Notably, Cui et al. demonstrated that RVG-conjugated EXOs derived from mesenchymal stem cells (MSC-RVG-Exos) improved targeting to the brain, reduced Aβ plaque deposition, and modulated inflammatory responses in APP/PS1 mice [49]. This pioneering work established the feasibility of RVG-mediated EXO delivery for AD. However, the mechanistic understanding was primarily centered on anti-inflammatory effects, and the specific molecular cargo responsible for therapeutic benefits was not fully elucidated. More recent studies have employed genetic engineering to generate EXOs displaying RVG peptide and simultaneously enriching therapeutic proteins such as neprilysin for Aβ degradation [56]. While these approaches demonstrate enhanced efficacy, they rely on complex genetic manipulation of parental cells and focus on exogenous cargo loading rather than harnessing the intrinsic therapeutic potential of the EXOs themselves. Compared with these existing approaches, our study offers several distinct advantages and significant advances. Firstly, we employed young human plasma-derived EXOs. This source is particularly advantageous because young plasma EXOs are naturally enriched with neuroprotective and “rejuvenating” factors, including the specific miRNAs (miR-23a-3p, miR-23b-3p, etc.) that were identified as key effectors targeting *RPTOR* in our study. This leverages the intrinsic therapeutic cargo of young plasma rather than relying on exogenous loading. Secondly, young plasma is more readily accessible and cost-effective compared to MSC culture supernatants or genetically modified cell systems, which require complex and expensive maintenance, transfection, and selection procedures. This practical advantage significantly enhances the translational potential of our strategy for clinical applications. Furthermore, we have elucidated a complete mechanistic pathway. While previous studies on RVG-engineered EXOs primarily focused on phenotypic outcomes, such as Aβ reduction and modulation of inflammatory responses, our study not only examined these phenotypic effects but also clarified the underlying mechanistic pathway.

The core innovations of this study are mainly reflected in the following three aspects. First, it is the first to demonstrate the direct application value of plasma-derived EXOs from young individuals in the treatment of AD. Although young plasma EXOs have been previously investigated in the context of anti-aging research, their role in ameliorating the pathological progression of AD has not been systematically elucidated. Through experiments in vitro and in vivo, this study provides the first validation of their therapeutic potential in AD models. Second, it elucidates the molecular mechanisms by which young plasma-derived EXOs exert their therapeutic effects through the RPTOR-autophagy axis. We identified that miRNAs enriched in plasma EXOs from young individuals, particularly miR-23a-3p and miR-23b-3p, directly target *RPTOR*. Functional experiments further confirmed that RVG-EXOs promote the clearance of pathological proteins by inhibiting *RPTOR* expression and activating the autophagy pathway. These findings elevate the therapeutic role of young plasma EXOs from phenomenological descriptions to the molecular pathway level. Third, RVG-engineered modification significantly enhanced the brain-targeting delivery efficiency and therapeutic efficacy of plasma-derived EXOs from young individuals. To address the limitation that natural EXOs are difficult to cross the BBB, this study successfully constructed RVG-EXOs and demonstrated their ability to significantly improve neuron-targeting ability and brain enrichment efficiency, thereby enhancing pathological improvement and cognitive protection in AD models. This provides an effective strategy for the targeted delivery of EXOs in CNS diseases.

Nevertheless, this study has several limitations. First, although we have established the critical role of the RPTOR-autophagy axis in mediating the therapeutic effects of RVG-EXOs, the potential involvement of other molecular pathways cannot be excluded and warrants further investigation. Second, the activation of autophagy is a “double-edged sword” (i.e., its long-term effects and safety in more advanced AD models need to be evaluated over longer observation periods). Finally, translating young plasma EXOs into clinical therapy requires addressing challenges such as large-scale standardized production, quality control, and ethical regulations. Future research should focus on: (1) optimizing engineering strategies to further enhance targeting efficiency; (2) exploring other youth-related active molecules; (3) combining RVG-EXOs with existing drugs to assess synergistic therapeutic effects on the treatment of AD; (4) and advancing relevant preclinical safety evaluations.

## 4 Conclusion

In summary, this study successfully developed an RVG-EXO formulation and demonstrated its ability to efficiently target the CNS. By inhibiting *RPTOR* expression, it activates autophagic flux, ultimately achieving comprehensive therapeutic outcomes in AD models, including alleviating pathology, protecting neurons, and improving cognitive function. This work not only provides a promising novel biological agent for the targeted treatment of AD but also opens a new mechanistic perspective on how youthful circulating factors modulate aging-associated disorders.

## 5 Materials and methods

### 5.1 Chemical and reagents

The RVG-29 was synthesized by Shenzhen Meluo Technology Co., Ltd. (Shenzhen, China). DSPE-PEG2000 was obtained from Shenzhen Meluo Technology Co., Ltd. (Shenzhen, China). Exosome Isolation and Purification Kit (from Plasma or Serum) Plus (UR52151) and the exosome-labeling dyes PKH67 (UR52303) and DiRm (UR21017) were obtained from Umibio (Shanghai) Co., Ltd. (Shanghai, China). Anti-CD63 antibody (A5271) was obtained from ABclonal Biotechnology Co., Ltd. (Wuhan, China). Anti-ALIX antibody (ab186429), Anti-P62 antibody (ab109012), Anti-phospho-Tau (Ser396) antibody (ab32057), Anti-neun antibody (AB104224), Anti-Synaptophysin antibody (AB32127), and Anti-MAP2 antibody (ab5392) were obtained from Abcam (Waltham, USA). Anti-Aβ antibody (15126), Anti-LAMP1 antibody (15665), and Anti-Raptor antibody (2280) were obtained from Cell Signaling Technology (Danvers, USA). LC3B-Specific Polyclonal antibody (18725-1-AP), Anti-GAPDH antibody (60004-1-lg), Anti-phospho-Tau (Ser202/Thr205) antibody (82568-1-RR), Anti-rabbit IgG (SA00001-2) antibody, Anti-mouse IgG (SA00001-1) antibody, and Anti-Beta Actin antibody (20536-1-AP) were obtained from Proteintech (Wuhan, China). Dulbecco's Modified Eagle Medium (DMEM) (PM150210, Procell, China), fetal bovine serum (FBS) (164210), and Minimum Essential Medium (PM150410) were obtained from Procell Life Science & Technology Co., Ltd. (Wuhan, China). Amyloid β peptide (1-42) human (P9001-5 mg, Beyotime, China), okadaic acid (S1786-10 μg), Calcein-AM/PI Cell Viability/Cytotoxicity Assay Kit (C1371S), RIPA Lysis Buffer (strong) (P0013B), and Western and IP Cell Lysis Buffer (P0013) were obtained from Beyotime Biotechnology (Shanghai, China). Lentiviral vectors for overexpressing *RPTOR* (human) and *Rptor* (mouse) were constructed by Shanghai GeneChem Co., Ltd. (Shanghai, China). 4% paraformaldehyde fixative (143174) was obtained from Biosharp Life Sciences (Beijing, China). Methanol was obtained from Tianjin Fuyu Fine Chemical Co., Ltd. (Tianjin, China). 20× Tris-EDTA Antigen Retrieval Buffer (pH 9.0) (G1203-250 ML), Antifade Mounting Medium with DAPI (G1407-25 ML), SWE Rapid High-Resolution Electrophoresis Buffer (powder) (G2081-1L), and Tris-Glycine Transfer Buffer (Powder) (G2017-1L) were obtained from Wuhan Servicebio Technology Co., Ltd. (Wuhan, China). Phosphatase Inhibitor Cocktail (100×) (GRF102) was obtained from Shanghai Yamoen Biomedical Technology Co., Ltd. (Shanghai, China). Triton X-100 (T8200), PMSF (P0100), and BCA Protein Assay Kit (PC0020) were obtained from Beijing Solarbio Science & Technology Co., Ltd. (Beijing, China). Mouse IL-6 ELISA Kit (EK0411), Mouse IL-1 beta/IL1B ELISA Kit (EK0394), and Mouse TNF Alpha/TNFA ELISA Kit (EK0527) were obtained from Boster Biological Technology Co., Ltd. (Pleasanton, USA)

### 5.2 Animals

Eight-month-old triple-transgenic AD (3×Tg-AD) mice [B6C3-Tg; (APPswe, Psen1M146V, tauP301L)/V, male] and their age- and sex-matched wild-type (WT) mice were purchased from Jinan Xingkang Biotech (Jinan, China) and housed in a temperature-controlled facility under a 12-h light/dark cycle with ad libitum access to sterile food and water. The animals were allowed to acclimate for 1 week prior to the initiation of experimental procedures, which were approved by the Animal Care and Use Committee of Shandong Provincial Hospital affiliated to Shandong First Medical University and were conducted following the institutional guidelines.

### 5.3 Isolation of exosomes from young human plasma

Healthy young donors were recruited through Shandong Provincial Hospital. All participants underwent a detailed health assessment prior to donation, including questionnaire surveys and medical report reviews. The inclusion criteria were healthy individuals aged between 18 and 25 years. The exclusion criteria included a diagnosis of cancer, HIV, COVID-19, any form of brain disease, or testing positive for hepatitis B or C surface antigens or antibodies. All donors provided written informed consent. Blood samples collected from donors were centrifuged at 1150 × g for 20 min to isolate plasma, which was then aliquoted into 500 μL portions and immediately stored at −80 °C for subsequent EXO extraction. EXOs were purified from 500 μL of plasma using the Exosome Isolation and Purification Kit (from Plasma or Serum) Plus (UR52151, Umibio Biotechnology, Shanghai, China), strictly following the manufacturer's instructions. The precipitation-based kit employed was inherently scalable and could be adapted for large-scale production by processing larger volumes of plasma or utilizing automated systems.

To minimize the impact of individual variability, plasma samples were collected from multiple healthy young donors (aged 18–25 years). EXOs were isolated separately from each donor's plasma, and equal amounts of EXOs from each donor were then pooled to obtain the final EXOs preparation. This pooling strategy has been widely adopted in EXO research to reduce batch effects and individual bias, thereby enhancing the reproducibility and generalizability of the experimental findings [57].

This study was approved by the Ethics Committee of the Provincial Hospital affiliated with Shandong First Medical University for Biomedical Research Involving Human Subjects (NSFC No. 2022–511). All procedures were conducted in accordance with the ethical principles of the Declaration of Helsinki. Written informed consent was obtained from all participants prior to enrollment.

### 5.4 RVG-engineered exosome production

RVG-EXOs were constructed by Shenzhen Meluo Technology Co., Ltd. (Shenzhen, China). First, the RVG-29 peptide and the lipid anchor DSPE-PEG2000 were mixed. Then, the solution was dried under a nitrogen stream to form a lipid film, which was subsequently hydrated with PBS (pH 7.4) in a 55 °C water bath. Finally, ultrasonication was applied to prepare the DSPE-PEG2000-RVG29 nanomicelle solution.

The extraction of EXOs from young human plasma was performed as previously described (see the “Exosome Isolation” section). The purified EXOs were mixed with the prepared DSPE-PEG2000-RVG29 nanomicelle solution and co-incubated at 37 °C with gentle agitation. Following incubation, the mixture was washed twice via ultracentrifugation to remove unbound micelles and free peptides. The final pellet was resuspended in sterile PBS to obtain RVG-EXOs, which were aliquoted and stored at −80 °C for future use.

### 5.5 Characterization of EXOs and RVG-EXOs

For morphological observation of EXOs and RVG-EXOs, the samples resuspended in PBS filtered through a 0.02-μm membrane were applied onto a 200-mesh carbon-coated copper grid and left to adsorb at room temperature for 5 min. Subsequently, excess liquid was carefully removed with filter paper, and the grid was negatively stained with uranyl acetate solution for 10 s. After drying at room temperature for 30 min, the samples were finally imaged using TEM (H7700, Hitachi, Japan).

To determine the size distribution and concentration of EXOs and RVG-EXOs, samples were diluted to 500 μL with PBS filtered through a 0.02-μm membrane. NTA was then performed using a NanoSight NS300 system (Malvern Instruments, UK). During detection, the NTA software (version 3.4, Build 3.4.4) was employed with the camera level set to 11 and the detection threshold set to 5 for data acquisition and analysis.

To measure the zeta potential of EXOs and RVG-EXOs, purified EXOs from young human plasma and RVG-EXOs were diluted in 1× PBS to a final concentration of approximately 1 × 10^9^ particles/mL. Measurements were performed at Shenzhen Meluo Technology Co., Ltd. (Shenzhen, China) using a ZetaPlus analyzer (Brookhaven Instruments Corporation, New York, USA). Each sample was loaded into a disposable clear zeta potential cuvette, equilibrated at 25 °C for 60 s, and then the absorbance was measured. Three independent measurements were conducted per sample, with each measurement consisting of 15 runs. The zeta potential values were calculated using the instrument's built-in Smoluchowski model. Data were presented as the mean ± standard deviation (SD) of the three independent experiments.

To quantitatively analyze the binding efficiency of RVG peptides on the EXO surface, nanoscale flow cytometry analysis was performed for EXO and RVG-EXO samples by Shenzhen Meluo Technology Co., Ltd. (Shenzhen, China). Briefly, samples were diluted in PBS to approximately 1 × 10^8^ particles/mL and incubated with an FITC-conjugated RVG antibody at 4 °C in the dark for 30–60 min, with an isotype control set in parallel. Unbound antibodies were then removed using a size-exclusion column, and the eluate was resuspended and calibrated with standard fluorescent nanoparticles. Finally, more than 10,000 particle events were acquired on a nanoflow cytometer (e.g., NanoFCM). The EXO population was gated based on side scatter, and the FITC fluorescence signal was analyzed to calculate the percentage of positive particles and the median fluorescence intensity, thereby assessing the RVG modification efficiency.

### 5.6 Tracking of EXOs and RVG-EXOs in vivo and in vitro

To monitor the in vivo biodistribution and cellular uptake of EXOs and RVG-EXOs, the EXOs were labeled with the lipophilic fluorescent dyes DiR (UR21017) and PKH67 (UR52303) obtained from Umibio Biotechnology (Shanghai, China) for in vivo and in vitro tracking experiments, respectively.

To evaluate the long-term in vivo distribution of EXOs and RVG-EXOs, purified EXOs were labeled by co-incubation with DiR dye at 37 °C in the dark for 30 min. The labeling reaction was terminated by adding an equal volume of PBS containing 1% BSA. Unbound dye was removed through a secondary purification step (either ultracentrifugation or size-exclusion chromatography). Six experimental mice received intravenous injections of 200 μg DiR-labeled EXOs or RVG-EXOs via the tail vein, while control mice were injected with free DiD dye. For three of the mice, the heart, liver, spleen, lungs, kidneys, and brain were collected 6 h after injection to capture fluorescence signals from these major organs. For the other three mice, whole-body fluorescence signals were collected at 6, 24, and 48 h post-injection using the IVIS Lumina II in vivo imaging system from Keygen BioTECH (Jiangsu, China).

To investigate the brain-targeting capability and cellular internalization of EXOs and RVG-EXOs, the EXOs were labeled with the PKH67 dye. The suspension of EXOs or RVG-EXOs was thoroughly mixed with the PKH67 dye by vortexing for 1 min, followed by incubation at 37 °C in the dark for 10 min. The staining was terminated by adding 1% BSA/PBS, and free dye was removed through a purification step. Mice were intravenously injected via the tail vein with 200 μg of PKH67-labeled EXOs or RVG-EXOs. In 4 h and 24h, brain tissues were collected and processed to make frozen sections for observation of EXO distribution using fluorescence microscopy.

To directly observe the cellular uptake of EXOs and RVG-EXOs, cultured neuronal cells were co-incubated with PKH67-labeled EXOs or RVG-EXOs under standard incubation conditions. After incubation, the culture medium was removed, and the cells were washed with PBS. Subsequently, the cells were fixed and examined under a fluorescence microscope to assess the internalization of EXOs or RVG-EXOs.

### 5.7 Animal Treatments

Two animal treatment plans were conducted.

*Plan A.* This study utilized 8-month-old 3xTg AD model mice (male), with their WT littermates serving as controls. All mice were randomly assigned to four groups (n = 8 per group): the Tg-Vehicle group (AD control), the WT-Vehicle group (WT control), the Tg-EXO group (treated with young plasma-derived EXOs), and the Tg-RVG-EXO group (treated with RVG-engineered EXOs). The protein concentrations of the young plasma-derived EXOs and RVG-EXOs were determined using a BCA protein assay kit (PC0020, Solarbio, Beijing, China). The administration concentration was selected based on previous studies [9], which demonstrated that EXOs isolated from young plasma, diluted with PBS to a total protein concentration of 1.8 μg/μL and administered via tail vein injection to aged mice, effectively improved cognitive function without causing significant toxicity to other organs. To ensure consistency with the effective dose reported in the literature, the working concentration of EXOs and RVG-EXOs in this study was set at 1.8 μg/μL. During the treatment, mice in the Tg-EXO and Tg-RVG-EXO groups received intravenous injections via the tail vein of 100 μL of EXOs or RVG-EXOs at a concentration of 1.8 μg/μL, administered three times per week for 4 consecutive weeks. The Tg-Vehicle and WT-Vehicle control groups were injected with an equal volume of PBS following the same dosing regimen.

*Plan B.* Eight-month-old male 3xTg mice were randomly divided into four groups (n = 8 per group): the Tg-EXO group (treated with EXOs), the Tg-RVG-EXO group (treated with RVG-EXOs), the Tg-RVG-EXO-NC group (injected with empty lentivirus and RVG-EXOs), and the Tg-RVG-EXO-OE group (injected with *Rptor*-overexpressing lentivirus and RVG-EXOs). The lentiviruses containing mouse *Rptor* (LV-gcGFP) and the empty lentivirus were provided by Shanghai GeneChem Co., Ltd. (Shanghai, China). Lentivirus with a titer of 1.5 × 10^9^ TU/mL were stereotactically injected into the hippocampus of mice at the following coordinates: AP, −2.0 mm; ML, ± 1.0 mm; DV, −2.0 mm [58], with a rate of 400 nL/min and a total volume of 1 μL unilaterally. The procedure for intravenous injection of EXOs or RVG-EXOs into mice was performed as described above in the treatment protocol of Plan A. Upon completion of behavioral testing, all mice underwent perfusion, and brain tissue samples were immediately collected for further analysis.

### 5.8 Morris Water Maze test

To assess spatial learning and memory abilities, the MWM test was performed on mice from each group, as previously reported [59]. Mice were first trained in a circular pool (diameter: 120 cm; water temperature: 22 ± 1 °C) that was divided into four quadrants with distinct visual cues. The experimental protocol consisted of an adaptation and visible platform session on day 1, followed by 5 consecutive days of hidden platform training (4 trials per day), and a probe test on day 7. On day 1 (visible platform session), the platform (10 cm in diameter), marked with a visible cue, was placed above the water surface for mice to identify. During days 2–6 (hidden platform navigation), the platform was fixed in the center of the target quadrant, submerged approximately 2 cm below the water surface. Each mouse underwent multiple trials daily. In each trial, the mouse was gently placed into the water and allowed to swim freely for a maximum of 60 s. If the mouse successfully located and mounted the platform, then it was allowed to remain there for 5 s before being removed. If the mouse failed to find the platform within 60 s, then it was gently guided to the platform and allowed to stay for 5 s. On day 7 (probe test), the platform was completely removed. Each mouse was introduced into the pool from the entry point in the quadrant opposite to the original platform location and allowed to swim freely for 60 s. An overhead tracking system was used to record escape latency and the number of platform crossings.

### 5.9 Y-maze test

The Y-maze test was employed to assess short-term spatial working memory in mice [60]. The Y-maze consisted of three identical arms (each of 40 cm in length, 15 cm in height, and 10 cm in width) arranged at 120° angles to each other, with interiors made of grey opaque material. Prior to testing, all mice (grouping consistent with the Plan A or Plan B of Animal Treatments) were placed in a quiet behavioral laboratory for a 30-min habituation period. During the formal test, a single mouse was placed in the central area and allowed to freely explore all three arms for a total of 5 min. The movement trajectory of the mouse was recorded using the ANY-maze video tracking system. The primary analytical metric was based on the Spontaneous Alternation Rate, defined as the percentage of actual alternations (consecutive entries into three different arms) relative to the maximum possible alternations (total arm entries minus 2). All experiments were conducted under consistent lighting, temperature, and humidity conditions at the same fixed time each day. The maze was thoroughly wiped with 75% ethanol after each mouse to eliminate odor cues.

### 5.10 Open Field Test

The OFT was conducted to evaluate autonomous exploratory behavior and the anxiety-like state in mice [61]. The open field apparatus contained a white square open chamber (length × width × height = 50 × 50 × 40 cm). The floor was divided by black grid lines into 16 squares of equal area, with the central region defined as a square area of 25 cm × 25 cm. The experiment was conducted in a quiet behavioral laboratory with uniform lighting. During testing, a single mouse was gently placed in the central square and allowed to explore freely for 10 min. A video tracking system was used to record the movement trajectory of the mouse throughout the session. Analyzed parameters included duration spent in the central area, number of entries into the central area, total distance traveled, and average movement speed. After each test, the interior walls and floor of the open field chamber were thoroughly cleaned with 75% ethanol to eliminate residual odors.

### 5.11 Novel object recognition test

The NOR test was employed to assess non-spatial recognition memory in mice [62]. The experimental apparatus contained a white square open field (length × width × height = 50 × 50 × 40 cm). Two identical objects that were difficult for mice to displace (e.g., building blocks or glass bottles, approximately 10 cm in height) were used for testing. The procedure consisted of three phases. (1) During the habituation phase (day 1), each mouse was placed individually into the empty apparatus and allowed to explore freely for 5 min (2) During the familiarization phase (24 h after the habituation phase), two identical objects were placed symmetrically in the apparatus, and the mouse was allowed to explore freely for 5 min (3) During the test phase (1 h after the familiarization phase), one of the two objects was replaced with a novel object (different in shape, color, or texture), and the mouse was again allowed to explore freely for 5 min. All sessions were recorded using a video tracking system, and active exploration behavior—defined as when the nose of the mouse was within 2 cm of an object—was analyzed with manual assistance. The primary outcome measure was the RI, calculated using the formula: [(time spent exploring the novel object)/(time spent exploring the novel object + time spent exploring the familiar object)] × 100%. After each test, the apparatus and objects were thoroughly cleaned with 75% ethanol to eliminate odor cues.

### 5.12 Nissl staining

To assess the survival of hippocampal neurons, Nissl staining was performed on brain tissue sections, as previously described [63]. After transcardial perfusion under anesthesia, the mouse brains were removed and fixed in 4% paraformaldehyde for 24 h. Following dehydration in a graded sucrose series, coronal sections of 30 μm thickness were prepared using a cryostat. Prior to staining, the sections were rinsed with 0.1 M PBS and then stained in 0.5% toluidine blue solution at 37 °C for 30 min in the dark. After staining, the sections were rapidly differentiated and dehydrated through a graded ethanol series (70%, 95%, and 100%), cleared in xylene, and mounted with neutral balsam. All sections were scanned in their entirety using a digital slide scanner. The number of neurons with clearly defined Nissl bodies was quantified using ImageJ software (National Institutes of Health, USA), with three non-consecutive sections randomly selected per mouse for counting.

### 5.13 Transmission electron microscopy analysis

TEM was employed to observe and quantitatively analyze the ultrastructure of autophagolysosomes in the hippocampal tissue of mice. Following cardiac perfusion, brain tissues were fixed in electron microscopy-grade fixative, post-fixed with 1% osmium tetroxide, dehydrated, and embedded in 812 resin. Ultrathin sections (60–80 nm thickness) were prepared, stained with uranyl acetate and lead citrate, and imaged using a Hitachi HT7800 TEM. Quantitative analysis of autophagolysosomes was performed using ImageJ software (National Institutes of Health, USA).

### 5.14 Golgi staining

To observe and analyze the morphology and density of neuronal dendritic spines, Golgi staining was performed on brain tissues, as previously reported [64]. Freshly obtained mouse brain tissues were immersed in Golgi staining solution (containing potassium dichromate, mercuric chloride, etc.) and impregnated in the dark for 14 d. The tissues were then transferred to a 30% sucrose solution until the samples sank to the bottom of the solution. Coronal sections of 150 μm thickness were prepared using a vibratome. Following dehydration through a graded ethanol series, the sections were cleared in xylene and mounted with neutral resin. All sections were observed under a light microscope to examine dendritic spine morphology. Dendritic spine density and morphology were quantitatively analyzed using ImageJ software (National Institutes of Health, USA).

### 5.15 Immunofluorescence staining of mouse brain tissues

The immunofluorescence staining was used to localize and detect specific antigens in brain tissue sections. The specific procedures were as follows: brain sections stored at −80 °C were retrieved and equilibrated to room temperature in a humidified chamber. The tissue area was circled with an immunohistochemistry pen, covered with PBS for 2 min, and then the PBS was removed. The sections were fixed with 4% paraformaldehyde at room temperature for 30 min and then washed three times (each of 5 min) with an adequate volume of PBS on a shaker. Antigen retrieval was then performed as follows: the sections were immersed in 1× EDTA retrieval buffer (pH 8.0) and heated in a microwave oven on medium power for 8 min, allowed to stand for 8 min, and then heated again on medium-low power for 7 min. After retrieval, the sections were cooled naturally to room temperature. Permeabilization was subsequently conducted by incubating with 0.5% Triton X-100 at room temperature for 10–15 min. The tissue area was re-circled with the pen, followed by three washes with PBS. The sections were blocked with 3% BSA blocking solution at room temperature for 1 h. After removing the blocking solution, the corresponding primary antibody working solution was applied, and the sections were incubated at 4 °C overnight. Then, the sections were washed three times with PBST. A species-matched fluorescent secondary antibody (diluted 1:500) was then applied, and the sections were incubated at room temperature for 1 h in the dark, followed by three washes with PBST. Finally, the sections were counterstained for nuclei by applying an antifade mounting medium containing DAPI, covered with a coverslip, protected from light, and observed under a confocal microscope for image acquisition. Fluorescence intensity and positive cell quantification were performed using ImageJ software (National Institutes of Health, USA). The primary antibodies used in this experiment included Anti-Aβ antibody (15126, Cell Signaling Technology, Danvers, USA), Anti-phospho-Tau (Ser202/Thr205) antibody (82568-1-RR, Proteintech, Wuhan, China), and Anti-P62 antibody (ab109012), Anti-neun antibody (AB104224), Alexa Fluor® 555 (ab150118), and Alexa Fluor® 488 (ab150081) obtained from Abcam (Waltham, USA).

### 5.16 Hematoxylin and eosin staining

Paraffin sections were dewaxed and rehydrated through a graded ethanol series, then the sections underwent staining with an hematoxylin and eosin (H&E) HD constant dye kit (G1076, Servicebio, China) and a Masson dye solution set (G1006, Servicebio, China) according to the manufacturer's instructions [65]. Stained sections were scanned using a light microscope (Nikon Eclipse 100), and histological features were evaluated by image analysis.

### 5.17 Cell culture

The human neuroblastoma SH-SY5Y cell line was used for all in vitro experiments. Cells were cultured in MEM medium supplemented with 10% fetal bovine serum (FBS) and 1% penicillin–streptomycin solution and maintained in a humidified incubator at 37 °C with 5% CO_2_. The cells grew adherently and were passaged every 2–3 d using 0.25% trypsin-EDTA solution when reaching 80–90% confluence [66]. All experiments were performed using cells in the logarithmic growth phase with a passage number of less than 30.

### 5.18 Cell treatments

Two treatment plans were applied to the cells.

*Plan A.* SH-SY5Y cells were pretreated with 20 μM Aβ1-42 for 48 h and subsequently co-cultured for 24 h with either PBS, EXOs, or RVG-EXOs. The group receiving PBS only was designated as the control. The dosage of EXOs and RVG-EXOs in cell experiments was determined based previous studied [9], which confirmed that incubating young plasma-derived EXOs with a total protein content of 50 μg with 1 × 10^6^ NE-4C or C2C12 cells effectively improved cellular function without inducing cytotoxicity. Accordingly, in this study, young plasma-derived EXOs or RVG-EXOs with a total protein content of 50 μg were incubated with 1 × 10^6^ SH-SY5Y cells for treatment.

*Plan B.* Untransfected SH-SY5Y cells were treated with 20 μM Aβ1-42 and then co-cultured with either EXOs or RVG-EXOs. SH-SY5Y cells transfected with either an empty lentivirus or an *RPTOR*-overexpressing lentivirus, after the same Aβ1-42 pretreatment as that in Plan A, were co-cultured with RVG-EXOs for 24 h.

### 5.19 Protein extraction and Western blot analysis

Protein samples were prepared by lysing cells or tissues with RIPA lysis buffer containing protease and phosphatase inhibitor cocktails, followed by centrifugation to collect the supernatant. Protein concentrations of the samples were determined using a BCA protein assay kit (PC0020, Solarbio, Beijing, China). Equal amounts of total protein were separated by 12% SDS-polyacrylamide gel electrophoresis and then transferred onto PVDF membranes (ISEQ00010, Merck Millipore, Shanghai, China) using a wet transfer method. The membranes were blocked with 5% skim milk in TBST at room temperature for 1 h, followed by incubation with corresponding primary antibodies at 4 °C overnight. GAPDH or β-actin was used as an internal reference protein for normalization. Horseradish peroxidase (HRP)-conjugated secondary antibodies, including anti-rabbit IgG (SA00001-2) and anti-mouse IgG (SA00001-1), were used for detection. The primary antibodies used in this experiment included Anti-CD63 antibody (A5271, Abclonal, Wuhan, China); Anti-Synaptophysin antibody (AB32127), Anti-ALIX antibody (ab186429), Anti-P62 antibody (ab109012), and Anti-phospho-Tau (Ser396) antibody (ab32057) obtained from Abcam (Waltham, USA); LC3B-Specific Polyclonal antibody (18725-1-AP), Anti-GAPDH antibody (60004-1-lg), and Anti-Beta Actin antibody (20536-1-AP) obtained from Proteintech (Wuhan, China); Anti-Raptor antibody (2280, Cell Signaling Technology, Danvers, USA).

### 5.20 Construction of *RPTOR*-overexpressing SH-SY5Y stable cell line

*RPTOR*-overexpressing SH-SY5Y stable cell lines were established by lentiviral transduction. Briefly, SH-SY5Y cells were seeded in 24-well plates at an appropriate density and cultured for 16–24 h to reach 20–40% confluence. For infection, 40 μL of 25× HiTransG P reagent and the calculated volume of lentivirus (corresponding to MOI = 20 for SH-SY5Y cells; virus volume = MOI × cell number/viral titer) were added to each well. After 16 h of incubation at 37 °C, the medium was replaced with fresh complete medium. At 72 h post-infection, when cell confluence reached 70–80%, puromycin selection was initiated at 3.5 μg/mL for 48 h, followed by maintenance in medium containing 1 μg/mL puromycin until stably transduced polyclonal populations were expanded. Parallel infections with empty vector lentivirus were performed as controls.

### 5.21 Immunofluorescence staining of cells

Immunofluorescence staining of cells was performed as follows: after discarding the culture medium, cells were washed three times with PBS (each of 5 min) and then fixed with 4% paraformaldehyde (1 mL per well for a 12-well plate format) at room temperature for 10 min, followed by three PBS washes. Subsequently, permeabilization was carried out using 0.1% Triton X-100 for 1 min, then cells were washed three times with PBS. Blocking was performed by applying 5% BSA blocking solution at room temperature for 0.5–2 h. After removing the blocking solution, cells were directly incubated with PBS-diluted primary antibody at 4 °C overnight. Then, the primary antibody was retrieved, and cells were washed three times with PBST. The corresponding fluorescent secondary antibody (diluted 1:500 in PBST) was then applied and incubated at room temperature for 1 h in the dark, followed by three PBST washes. Finally, cell nuclei were counterstained by applying an antifade mounting medium containing DAPI. A coverslip was placed over the sample and sealed with nail polish. Imaging was performed using a confocal microscope. Fluorescence intensity and positive cell quantification were performed using ImageJ software (National Institutes of Health, USA). The primary antibodies used in this experiment included Anti-P62 antibody (ab109012), Anti-neun antibody (AB104224), Anti-MAP2 antibody (ab5392), Alexa Fluor® 488 (ab150081), and Alexa Fluor® 555 (ab150118) obtained from Abcam (Waltham, USA); Anti-LAMP1 antibody (15665, Cell Signaling Technology, Danvers, USA); and LC3B-Specific Polyclonal antibody (18725-1-AP, Proteintech, Wuhan, China).

### 5.22 Cell viability assay by Calcein-AM/PI double staining

Cell viability was assessed using the Calcein-AM/PI double-staining assay [67]. Briefly, SH-SY5Y cells were pre-treated with 20 μM Aβ1-42 for 48 h and then co-cultured with different treatment reagents for 24 h. Then, the cells were collected by centrifugation at 1000 × g for 5 min in a 24-well plate. The detection working solution was prepared by mixing 1 μL of Calcein-AM and 1 μL of PI into 1 mL of assay buffer. Then, 250 μL of the working solution was added to each well, followed by incubation at 37 °C in the dark for 30 min. Then, cells were observed and photographed under a fluorescence microscope using the green and red channels, respectively. Cell survival rate was assessed by counting the green (live cells) and red (dead cells) fluorescent signals.

### 5.23 Assessment of autophagic flux by tandem fluorescent LC3b reporter

To monitor autophagic flux dynamics, the tandem fluorescent-tagged autophagy reporter plasmid pmCherry-EGFP-LC3B (Research Cloud Biology, Jinan, China) was transfected into SH-SY5Y cells using Lipofectamine 3000 transfection reagent according to the manufacturer's instructions. Following transfection, the punctate accumulation of yellow fluorescence (representing autophagosomes) and red fluorescence (representing autolysosomes) was observed under a fluorescence microscope to assess the progression of autophagic flux. The fluorescent puncta within cells were counted and quantitatively analyzed using ImageJ software (National Institutes of Health, USA).

### 5.24 Assessment of Aβ clearance in SH-SY5Y cells

To evaluate the Aβ clearance capacity of SH-SY5Y cells, fluorescein-labeled 647-Aβ1-42 was used for tracing analysis [28]. The specific steps were as follows: SH-SY5Y cells were co-incubated with 2.5 μM Aβ1-42 and EXOs from different treatment groups (EXOs or RVG-EXOs) for 24 h. Subsequently, the cells were washed three times with PBS, and the medium was replaced with fresh medium containing 2 μg/mL (approximately 0.4 μM) 647-Aβ1-42 for an additional 3-h incubation. Then, the medium containing the fluorescently labeled Aβ was removed and replaced with fresh DMEM, followed by a 24-h culture to simulate the intracellular degradation and clearance of Aβ. Finally, the medium was discarded, the cells were washed three times with PBS, and subsequent cell staining and fluorescence imaging analysis were performed. The fluorescence signal intensity was quantified using ImageJ software (National Institutes of Health, USA) to assess the level of residual intracellular Aβ.

### 5.25 Assessment of P-Tau clearance in SH-SY5Y cells

To evaluate the clearance effect of EXOs on P-Tau, cells were seeded in 6-well plates at a density of 2 × 10^5^ cells per well and allowed to adhere overnight. To induce Tau protein hyperphosphorylation, cells were first treated with 40 nM OA for 24 h. Subsequently, the OA-containing medium was replaced with fresh medium containing either EXOs or RVG-EXOs for a 24-h co-culture. The untreated cells were used as a negative control, OA-treated cells alone as a positive control, and experimental groups contained OA-treated cells co-cultured with either EXOs or RVG-EXOs. After the co-culture period, the levels of the corresponding P-Tau (Ser396) were detected by Western blotting analysis.

### 5.26 Dual-luciferase reporter assay

The interaction of hsa-miR-23a-3p and hsa-miR-23b-3p with the 3′ UTR of *RPTOR* was predicted using the TargetScan database (https://www.targetscan.org/vert_80/). Based on the predicted binding site, WT and seed region-mutated (MUT) fragments of *RPTOR* were cloned into the pmirGLO vector (GenePharma, China). HEK-293T cells were seeded in 24-well plates and transfected with 500 ng of RPTOR-WT or RPTOR-MUT reporter plasmid, along with 50 nM hsa-miR-23a-3p mimic, hsa-miR-23b-3p mimic, or negative control mimic (GenePharma, China) using Lipofectamine 3000. After 48 h, luciferase activity was measured using the Dual-Luciferase® Reporter Assay System (11402ES60, YEASEN, China). Firefly luciferase activity was normalized to Renilla luciferase activity. All experiments were performed in triplicate, and results were presented as the mean ± standard deviation (SD).

### 5.27 RNA isolation and quantitative real-time PCR

Total RNA from cells and tissues was extracted using TRIzol reagent (AG21101, Accurate Biotechnology, China) and exosomal RNA was isolated with the ExoRNeasy Serum/Plasma Maxi Kit (Qiagen, Frankfurt, Germany). cDNA synthesis was performed using Evo M-MLV RT Premix (AG11706, Accurate Biotechnology, China) for mRNA and a miRNA First Strand cDNA Kit (Stem-loop) (AG11742, Accurate Biotechnology, China) for miRNA. Quantitative real-time PCR (qRT-PCR) was carried out using the SYBR® Green Pro Taq HS Premixed qPCR Kit (AG11701, Accurate Biotechnology, China) with gene-specific primers. *GAPDH* was used as internal control for mRNA normalization; *U6* and *cel-miR-39-3p* were used as internal and external controls for miRNA normalization, respectively. Relative expression was calculated using the 2^–ΔΔCT^ method. Primer sequences were listed in Table S1.

### 5.28 Cell transfection

For cell transfection experiments, 50 pmol of miRNA inhibitor (GenePharma, China) was transfected into SH-SY5Y cell line using Lipofectamine 3000 (L3000015, Thermo Fisher Scientific, USA) following the manufacturer's instructions [68]. Total RNA and protein were extracted 48 h after transfection. The sequence of has-miR-23a-3p inhibitor was 5′-GGAAAUCCCUGGCAAUGUGAU-3′, and the sequence of has-miR-23b-3p inhibitor was 5′-GUGGUAAUCCCUGGCAAUGUGAU-3’.

### 5.29 Enzyme-linked immunosorbent assay (ELISA)

The concentrations of interleukin-6 (IL-6), tumor necrosis factor-alpha (TNF-α), interleukin-1 beta (IL-1β), and immunoglobulin G (IgG) in mouse samples were measured using commercial ELISA kits according to the manufacturer's instructions. Plasma samples were collected and stored at −80 °C until analysis. For tissue samples, brain and liver tissues were homogenized in ice-cold PBS containing protease inhibitors, followed by centrifugation to collect the supernatants for protein quantification. The following kits from Boster Biological Technology Co., Ltd. (Pleasanton, USA) were utilized: Mouse IL-6 ELISA Kit (catalog number: EK0411), Mouse IL-1 beta/IL1B ELISA Kit (Cat. No.: EK0394), Mouse TNF Alpha/TNFA ELISA Kit (Cat. No.: EK0527), and Mouse IgG ELISA Kit (Cat. No.: EK0101). All assays were performed with three replicates. The optical density was measured at 450 nm using a microplate reader. The concentrations of the target proteins were calculated by comparing the sample optical densities with the standard curves.

### 5.30 Small RNA sequencing and analysis

Samples were obtained from six randomly selected elderly donors (aged ≥ 75 years), six donors with AD, and six young donors (aged ≤ 30 years). Total RNA was extracted from isolated exosomes, and sequencing libraries were prepared using the Multiplex Small RNA Library Prep Set for Illumina® (San Diego, CA, USA) following the manufacturer's protocol. Sequencing was conducted by Novogene Bioinformatics Technology Co., Ltd. (Beijing, China). Clean reads were filtered based on length distribution to enrich for small RNAs (sRNAs). Reads were aligned to the human reference genome (GRCh38) using Bowtie. Known miRNAs were annotated by alignment to miR Base v20.0, and novel miRNAs were predicted using a combination of miR Evo and miRDeep2. Differential expression analysis between the elderly and young groups was performed using the DESeq R package, and miRNAs with a log_2_ (fold change) > 1 and adjusted p–value (FDR) < 0.05 were considered significantly differentially expressed.

### 5.31 Clinical and demographic characterization of plasma donors

Samples were obtained from six randomly selected elderly donors (aged ≥ 75 years), six donors with AD, and six young donors (aged ≤ 30 years). Demographic details of the participants were provided in Table S2.

Peripheral blood was collected from each participant and placed into EDTA-coated anticoagulant tubes and processed within 4 h of collection. Plasma was separated by centrifugation at 1150 × g for 15 min, aliquoted into 500 μL portions, and stored at −80 °C until analysis. For Alzheimer's disease-related biomarker measurement, frozen plasma samples were thawed at 4 °C and then centrifuged at 10,000 × g for 10 min to remove residual cell debris. The resulting supernatant was then divided into 75 μL aliquots for quantification of Alzheimer's disease-related biomarkers using a single-molecule detection system (AXL-2000, Lychix, Suzhou, China).

This study was approved by the Ethics Committee of the Provincial Hospital affiliated with Shandong First Medical University for Biomedical Research Involving Human Subjects (NSFC No. 2022–511). All procedures were conducted in accordance with the ethical principles of the Declaration of Helsinki. Written informed consent was obtained from all participants prior to enrollment.

### 5.32 Single-nucleus RNA sequencing (snRNA-seq)

To investigate the transcriptional landscape of brain tissues following RVG-EXO treatment, 8-month-old 3xTg mice were intravenously injected (tail vein) with either RVG-EXOs (1.8 μg/μL, 100 μL per injection, three times per week for 4 weeks) or an equal volume of PBS (vehicle control) according to the treatment protocol described in Plan A (n = 3 mice per group). At the end of the treatment period, mice were euthanized, and whole brains were rapidly dissected. The hippocampal and cortical regions were isolated, flash-frozen in liquid nitrogen, and stored at −80 °C until further processing.

From fresh frozen mouse brain tissue, approximately 500 mg of tissue was cut and weighed on dry ice using sterile disposable scalpels. The brain tissue was homogenized in ice-cold homogenization buffer (0.25 M sucrose, 5 mM CaCl_2_, 3 mM MgAc_2_, 10 mM Tris-HCl pH 8.0, 0.1 mM EDTA, 1×protease inhibitor, and 1 U/μL Ribolock RNase inhibitors) with a glass-on-glass Dounce homogenizer: 10 strokes with the A pestle, followed by 10 strokes with the B pestle. Homogenates were passed through a 70-μm cell strainer to collect the nuclear fraction. The nuclear fraction was mixed with an equal volume of 50% iodixanol and added on top of a 30–33% iodixanol solution, then centrifuged for 20 min at 10,000×g, 4 °C. After removal of the myelin layer from the top of the gradient, the nuclei were collected from the 30–33% iodixanol interface. The nuclei were resuspended in nuclei wash and resuspension buffer (0.04% bovine serum albumin, 0.2 U/μL Ribolock RNase inhibitors, 500 mM mannitol, and 0.1 mM PMSF protease inhibitor in phosphate-buffered saline) and pelleted for 5 min at 500×g, 4 °C. The nuclei were passed through a 40-μm cell strainer to remove cell debris and large clumps. Nucleus concentration was manually determined using trypan blue counterstaining and a hemocytometer. The concentration was adjusted to 700–1200 nuclei/μL, and the nuclei were immediately processed following the 10x Genomics® Single Cell Protocol.

Single-nucleus libraries were constructed using the DNBelab C Series High-throughput Single-cell RNA Library Preparation Set V3.0 (TaiM 4, MGI, Shenzhen, China) following the manufacturer's protocol. Briefly, the nuclei suspension was loaded onto a DNBelab C4 single-cell instrument for droplet generation and barcoding. After reverse transcription, cDNA was recovered and amplified by PCR. The amplified cDNA was then used to construct both a cDNA library and an oligo library. The libraries were sequenced on a DNBSEQ-T7RS platform (MGI, Shenzhen, China) in paired-end 150 bp (PE150) mode.

Raw sequencing data were processed using dnbc4tools (version 2.1.0) for quality control, alignment to the mouse reference genome (mm10), and gene expression quantification. The resulting cell-by-gene matrix was analyzed using the Seurat package (version 4.3.0). Cells with low-quality metrics (e.g., high mitochondrial gene percentage, abnormal UMI or gene counts) and potential doublets were filtered out. Data were normalized and scaled, followed by principal component analysis (PCA), batch effect correction with Harmony, and cell clustering using the Louvain algorithm. Clusters were visualized by t-SNE and annotated using the SingleR R package. The snRNA-seq experiment and data analysis were performed by Genedenovo Biotechnology Co., Ltd. (Guangzhou, China).

### 5.33 Detection of APP and CD74 protein in vitro and in vivo

In cell experiments, human SH-SY5Y neurons were co-cultured with HMC3 microglia, and the following four treatment groups were established: (1) Negative control group, treated with PBS only; (2) Cu^2+^ control group, treated with 1 μM Cu^2+^ for 3 days (a condition previously confirmed to significantly upregulate APP protein levels in SH-SY5Y cells [69]), followed by incubation with PBS for 24 h; (3) RVG-EXOs alone group, treated with Cu^2+^ for 3 days, then incubated with 50 μg RVG-EXOs (per 1 × 10^6^ cells) for 24 h; (4) Combination treatment group, treated with Cu^2+^ for 3 days, then co-incubated with 50 μg RVG-EXOs and 5 mM 3-methyladenine (3-MA, a well-known autophagy inhibitor) for 24 h (this concentration and treatment duration of 3-MA have been shown to suppress cellular autophagy [70]). PBS was used as the solvent control in all cell experiments.

In the in vivo study, 8-month-old 3xTg mice were injected via the tail vein with 100 μL of RVG-EXOs at a concentration of 1.8 μg/μL, three times per week for two weeks. In addition, another group of mice received both the above tail-vein injections of RVG-EXOs and intraperitoneal injections of 3-MA at a dose of 30 mg/kg (a dose that has been shown to significantly suppress autophagy activity in the mouse brain [71]) at the same frequency. Control mice were injected with an equal volume of PBS.

After the respective treatments, cells and brain tissues were either fixed or lysed, and then processed for immunofluorescence staining and Western blot analysis following standard protocols. The reagents used included APP (E4H1U) Rabbit Monoclonal Antibody (76600, Cell Signaling Technology, Danvers, USA); CD74 Antibody (MU601117, Abmart, Shanghai, China); and Alexa Fluor® 488 anti-mouse CD74 (CLIP) Antibody (151005, BioLegend, San Diego, USA); Goat Anti-Rabbit IgG H&L (Alexa Fluor® 555) preadsorbed (ab150086, abcam, Waltham, USA); Goat Anti-Mouse IgG H&L (Alexa Fluor® 488) (ab150113, abcam, Waltham, USA); 3-Methyladenine (HY-19312, MedChemExpress LLC, New Jersey, USA).

### 5.34 Detection of excitatory neurons, inhibitory neurons, and DAM

For in vivo immunofluorescence assessment of neuronal and microglial subtypes, 8-month-old 3xTg mice received tail-vein injections of 100 μL RVG-EXOs (1.8 μg/μL) or an equivalent volume of PBS three times per week for two consecutive weeks. Subsequently, brain tissues were harvested and processed for immunofluorescence staining. Excitatory neurons were identified as NeuN^+^CaMK2a^+^, inhibitory neurons as NeuN^+^GAD1^+^, and DAM as IBA1^+^CLEC7A^+^. The primary antibodies used for this detection included CaMK2 alpha Polyclonal antibody (13730-1-AP, Proteintech, Wuhan, China), GAD1 Monoclonal antibody (67648-1-Ig, Proteintech, Wuhan, China), Dectin-1/Clec7a (E3P5W) Rabbit Monoclonal Antibody (30260, Cell Signaling Technology, Danvers, USA), Iba1 Mouse IgG1 mAb (OB-MMS039-02, Oasis biofarm, Zhejiang China), Goat Anti-Rabbit IgG H&L (Alexa Fluor® 555) preadsorbed (ab150086, abcam, Waltham, USA); Goat Anti-Mouse IgG H&L (Alexa Fluor® 488) (ab150113, abcam, Waltham, USA), Goat Anti-Rabbit IgG H&L (Alexa Fluor® 488) preadsorbed (ab150081, abcam, Waltham, USA), Goat Anti-Mouse IgG H&L (Alexa Fluor® 555) preadsorbed (ab150118, abcam, Waltham, USA), Anti-NeuN antibody (ab104224, abcam, Waltham, USA), Anti-NeuN antibody (ab177487, abcam, Waltham, USA). All stained sections were examined under a fluorescence microscope, and images were captured for quantitative analysis.

### 5.35 Statistical analyses

Bioinformatic analysis of single-cell RNA-sequencing data was performed using OmicsMaster, a dynamic real-time interactive online platform (https://report.omicsmaster.com). All other statistical analyses were performed using GraphPad Prism version 10.2 (RRID: SCR_002798). Data were presented as mean ± standard deviation (SD) unless otherwise specified. Comparative analyses between two groups were conducted to reveal the significant differences using unpaired two-tailed Student's t-tests. Comparative analyses among multiple groups were performed using one-way or two-way ANOVA followed by appropriate post hoc tests. Statistically significant difference was determined based on *P* < 0.05.

## CRediT authorship contribution statement

**Hang Chen:** Conceptualization, Data curation, Formal analysis, Investigation, Writing – original draft, Writing – review & editing. **Yuanquan Si:** Conceptualization, Resources, Software. **Qian Cheng:** Conceptualization, Investigation, Methodology. **Qian Yu:** Conceptualization, Methodology, Validation. **Zhikang Cui:** Conceptualization, Supervision. **Shuyi Yu:** Formal analysis, Investigation. **Xiaoyi Zhao:** Investigation, Methodology. **Yan Jin:** Data curation, Investigation, Methodology. **Yunshan Wang:** Data curation, Formal analysis, Methodology, Project administration. **Ming Li:** Conceptualization, Data curation, Formal analysis, Investigation, Methodology. **Zhiming Lu:** Conceptualization, Data curation, Formal analysis, Funding acquisition, Investigation, Supervision.

## Data and materials availability

All data are available in the main text or the supplementary materials.

## Ethics approval and consent to participate

Human studies were conducted in accordance with the Declaration of Helsinki and approved by the Ethics Committee of Shandong Provincial Hospital, affiliated with Shandong First Medical University (approval No. 2022-511). Written informed consent was obtained from all participants. All animal experiments were reviewed and approved by the Laboratory Animal Management and Ethics Review Committee of Shandong Provincial Hospital (approval No. 2022-511).

## Funding

This work was supported by the 10.13039/501100001809National Natural Science Foundation of China (82502804), the 10.13039/501100007129Natural Science Foundation of Shandong Province (ZR2025QC1673), and the Taishan Scholars Program of Shandong Province (tsqnz20240852).

## Declaration of competing interest

The authors declare no conflict of interest.
