# Supplementary material for: Therapeutic potential and underlying mechanisms of engineered young plasma-derived exosomes in Alzheimer's disease
Source: Bioact Mater. 2026 Aug 15;67:497–523. doi: 10.1016/j.bioactmat.2026.08.008 (PMC13499491; doi:10.1016/j.bioactmat.2026.08.008)
Supplement: Supplementary file 1 [file mmc1.docx]

**Fig. S1. Characterization and in vivo biodistribution of EXOs and RVG-EXOs. (A)** Quantitative analysis of the mean fluorescence intensity (MFI) of FITC in EXOs and RVG-EXOs (n = 3). **(B)** Western blot analysis of the negative markers GM130 and Calnexin in EXOs from different groups. **(C)** Representative in vivo images of mice from different treatment groups at 6, 24, and 48 h, along with ex vivo images of major organs at 6 h and quantitative analysis of EXOs and RVG-EXOs in the brain (n = 3). **(D)** Representative immunofluorescence images and quantitative assessment of uptake of EXOs and RVG-EXOs by neurons in brain tissue (n = 3). Data are presented as mean ± SD (n = 3). Statistical significance is determined by the t-test. Significance level: p < 0.05 (*), p < 0.01 (**), p < 0.001 (***), p < 0.0001 (****).

**Fig. S2. Neuroprotective effects of engineered EXOs. (A)** Representative immunofluorescence images of MAP2 in SH-SY5Y cells under different treatments. Scale bar = 100 μm. Blue, DAPI staining nuclei; green, MAP2. **(B)** Western blot analysis and quantification of synaptophysin in mouse brain tissue under different treatments (n = 3). Data are presented as mean ± SD (n = 3). Statistical significance is determined by the t-test. Significance level: p < 0.05 (*), p < 0.01 (**).

**Fig. S3. Engineered EXOs promote the expression of LC3B protein in neurons. (A)** Representative immunofluorescence images and quantitative analysis of LC3B in SH-SY5Y cells under different treatments (n = 3). Scale bar = 100 μm. Blue, DAPI staining nuclei; red, Neun; green, LC3B. Data are presented as mean ± SD (n = 3). Statistical significance is determined by the t-test. Significance level: p < 0.05 (*), p < 0.01 (**), p < 0.001 (***).

**Fig. S4. Engineered EXOs promote the expression of LC3B protein primarily by inhibiting the expression of the *RPTOR* gene. (A)** Dual-luciferase reporter assay confirming *RPTOR* as the target gene of miR-23a-3p and miR-23b-3p. **(B)** Quantitative qPCR analysis showing miR-23a-3p and miR-23b-3p expression levels in cells under different treatments (n = 3). **(C)** Western blot analysis of Raptor under different treatment conditions and its quantification **(D)** (n = 3). **(E–J)** Quantitative qPCR analysis showing miR-18a-3p, miR-23a-3p, miR-23b-3p, miR-4286, miR-4508, and miR-766-3p expression levels in EXOs and RVG-EXOs (n = 3). **(K)** Representative immunofluorescence images of LC3B protein in SH-SY5Y cells under different treatments and its quantitative analysis (n = 3). Green, LC3B; red, Neun; blue, DAPI staining nuclei. Scale bar = 100 μm. Data are presented as mean ± SD (n = 3) . Statistical significance is determined by the t-test. Significance level: ns, not significant; p < 0.05 (*), p < 0.01 (**), p < 0.001 (***), p < 0.0001 (****).

**Fig. S5. Engineered EXOs improve cognitive behavior in mice primarily via inhibiting the expression of the *RPTOR* gene. (A)** Western blot analysis and quantification of Raptor protein expression levels in the cortical tissue of mouse brain across different treatment groups (n = 3). **(B)** Western blot analysis and quantification of Raptor protein in the cortical tissue of mouse brain (n = 3). The results show the protein expression levels of Raptor in the cortical region of 3xTg mice after intervention via stereotaxic injection of an *RPTOR*-overexpressing lentivirus (Tg-RPTOR-OE) or an empty lentivirus control (Tg-RPTOR-NC). **(C)** Representative trajectory plots from the Y-maze test and quantitative analysis of the spontaneous alternation rate in mice under different treatments (n = 8). **(D)** Quantitative analysis of the Recognition Index across experimental groups of mice. Data are presented as mean ± SD. Statistical significance is determined by the t-test. Significance level: ns, not significant; p < 0.05 (*), p < 0.01 (**).

**Fig. S6. Correlation heatmap between Seurat clusters and SingleR cell type annotations. (A)** Correlation heatmap between Seurat clusters and SingleR cell type annotations. The heatmap shows the correspondence between Seurat clusters (rows, clusters 0-29) and SingleR-annotated cell types (columns). Color intensity represents the correlation score of cells within each Seurat cluster assigned to each cell type. Dark red indicates high correspondence, while blue indicates low correspondence.

**Fig. S7. Heatmap of cell-type proportions across clusters. (A)** The heatmap displays the proportion of various cell types (x-axis) across all identified clusters (y-axis, Cluster 0–29). The numeric value within each cell represents this proportion, and the color gradient from white (0) to dark red (1) indicates the scale from low to high.

**Fig. S8. UMAP clustering and cell type annotation of single-cell sequencing data. (A)** UMAP projection of single-cell transcriptomic data. Left panel shows unsupervised Seurat clusters (clusters 0-29). Right panel shows cell type annotations assigned by SingleR, including Neurons, Astrocytes, B cells, Endothelial cells, Epithelial cells, Fibroblasts, Granulocytes, Macrophages, Microglia, Monocytes, Oligodendrocytes, T cells.

**Fig. S9. Quantitative analysis of mean fluorescence intensity of different proteins. (A)** Quantitative analysis of mean fluorescence intensity of Camk2a protein in mouse brain tissues from different treatment groups (n = 3). **(B)** Quantitative analysis of mean fluorescence intensity of GAD1 protein in mouse brain tissues from different treatment groups (n = 3). **(C)** Quantitative analysis of mean fluorescence intensity of APP protein in cells from different treatment groups (n = 3). **(D)** Quantitative analysis of mean fluorescence intensity of CD74 protein in cells from different treatment groups (n = 3). **(E)** Quantitative analysis of mean fluorescence intensity of APP protein in mouse brain tissues from different treatment groups (n = 3). **(F)** Quantitative analysis of mean fluorescence intensity of CD74 protein in mouse brain tissues from different treatment groups (n = 3). **(G)** Quantitative analysis of mean fluorescence intensity of CLEC7A protein in mouse brain tissues from different treatment groups (n = 3). Data are presented as mean ± SD. Statistical significance is determined by the Student’s t-test based on *P* < 0.05 (*), *P* < 0.01 (**), and *P* < 0.001 (***); ns, not significant.

**Fig. S10. Biosafety evaluation of RVG-EXOs and EXOs in vivo. (A–C)** Quantitative ELISA analysis of the inflammatory cytokines IL-6, TNF-α, and IL-1β in the plasma of mice from different treatment groups. **(D)** ELISA quantification of IgG in plasma from different treatment groups of mice. **(E)** Representative HE staining images of the heart, liver, spleen, lung, kidney, and brain of mice from different treatment groups. **(F–H)** ELISA quantification of the inflammatory cytokines IL-6, TNF-α, and IL-1β in mouse liver tissue across different treatment groups. **(I–K)** ELISA quantification of the inflammatory cytokines IL-6, TNF-α, and IL-1β in mouse brain tissue across different treatment groups.

**Supplementary Table 1.** Primers and their sequences using in this study.

| **Gene** | **Primer sequence** |
| --- | --- |
| *RPTOR* | F: 5'-AGTGTGAGCGTCAATGGAGAT-3’  R: 5'-TTGTAGATGGCGGTGAACTGA-3' |
| *GAPDH* | F: 5′- AGGTCGGTGTGAACGGATTTG-3′ R: 5′- TGTAGACCATGTAGTTGAGGTCA-3′ |
| *U6* | F: 5′- GTGCAGGGTCCGAGGT -3′ R: 5′-TCTCTCTCTCTCTCTCTCTC-3′ |
| hsa-miR-18a-3p | F: 5′-CGACTGCCCTAAGTGCTCC-3′  R: 5′-AGTGCAGGGTCCGAGGTATT-3′ |
| hsa-miR-23a-3p | F: 5′-TCGGATCACATTGCCAGGG-3′  R: 5′-AGTGCAGGGTCCGAGGTATT-3′ |
| hsa-miR-23b-3p | F: 5′-TCGATCACATTGCCAGGGAT-3′  R: 5′-AGTGCAGGGTCCGAGGTATT-3′ |
| hsa-miR-4286 | F: 5′-GTCATGCACCCCACTCCT-3′  R: 5′-AGTGCAGGGTCCGAGGTATT-3′ |
| hsa-miR-4508 | F: 5′-GTTTATTGCGGGGCTGGG-3′  R: 5′-AGTGCAGGGTCCGAGGTATT-3′ |
| hsa-miR-766-3p | F: 5′-CGTTACTCCAGCCCCACAG-3′  R: 5′-AGTGCAGGGTCCGAGGTATT-3′ |
| cel-miR-39-3p | F: 5′-GGCGACCGGGTGTAAATCA-3′  R: 5′-AGTGCAGGGTCCGAGGTATT-3′ |

**Supplementary Table 2. Clinical and demographic characterization of plasma donors**

| **Characteristics** | **编号** | **Age (year)** | **Gender** | **Plasma** **P-Tau217 (pg/ml)** | **Plasma GFAP (pg/ml)** | **Plasma NFL (pg/ml)** | **Plasma Aβ42/40** | **MMSE** |
| --- | --- | --- | --- | --- | --- | --- | --- | --- |
| **Young** | 01 | 19 | Male | 0.66 | 19.62 | 5.91 | 0.079 | 30 |
|  | 02 | 22 | Male | 0.51 | 26.11 | 6.94 | 0.081 | 30 |
|  | 03 | 23 | Female | 0.94 | 29.03 | 8.05 | 0.073 | 30 |
|  | 04 | 19 | Male | 0.86 | 33.87 | 7.38 | 0.097 | 29 |
|  | 05 | 25 | Female | 0.53 | 38.35 | 8.29 | 0.093 | 29 |
|  | 06 | 20 | Male | 0.98 | 34.92 | 7.73 | 0.086 | 30 |
| **AD** | 07 | 77 | Male | 1.96 | 123.94 | 44.15 | 0.027 | 22 |
|  | 08 | 79 | Male | 2.03 | 138.37 | 49.38 | 0.022 | 21 |
|  | 09 | 80 | Male | 2.66 | 153.87 | 50.04 | 0.029 | 18 |
|  | 10 | 77 | Female | 2.51 | 162.85 | 51.27 | 0.046 | 19 |
|  | 11 | 78 | Female | 2.79 | 144.72 | 41.33 | 0.033 | 15 |
|  | 12 | 82 | Male | 3.11 | 189.22 | 55.08 | 0.031 | 18 |
| **Elderly** | 13 | 76 | Male | 0.82 | 44.31 | 21.95 | 0.066 | 28 |
|  | 14 | 78 | Female | 0.99 | 56.09 | 29.76 | 0.059 | 29 |
|  | 15 | 81 | Female | 1.21 | 59.26 | 31.69 | 0.062 | 28 |
|  | 16 | 79 | Male | 1.06 | 49.76 | 23.11 | 0.072 | 28 |
|  | 17 | 78 | Female | 0.94 | 63.08 | 25.54 | 0.088 | 29 |
|  | 18 | 83 | Male | 1.33 | 68.97 | 36.07 | 0.051 | 27 |
